# A Novel OxyR Sensor and Regulator of Hydrogen Peroxide Stress with One Cysteine Residue in *Deinococcus radiodurans*

**DOI:** 10.1371/journal.pone.0001602

**Published:** 2008-02-13

**Authors:** Huan Chen, Guangzhi Xu, Ye Zhao, Bing Tian, Huiming Lu, Xiaomin Yu, Zhenjian Xu, Nanjiao Ying, Songnian Hu, Yuejin Hua

**Affiliations:** 1 Key Laboratory of Chinese Ministry of Agriculture for Nuclear-Agricultural Sciences, Institute of Nuclear-Agricultural Sciences, Zhejiang University, China; 2 James D. Watson Institute of Genome Sciences, Zhejiang University, Hangzhou, China; 3 Key Laboratory of Genome Science and Information, Beijing Institute of Genomics, Chinese Academy of Sciences, Beijing, China; University of Munich and Center of Integrated Protein Science, Germany

## Abstract

In bacteria, OxyR is a peroxide sensor and transcription regulator, which can sense the presence of reactive oxygen species and induce antioxidant system. When the cells are exposed to H_2_O_2_, OxyR protein is activated via the formation of a disulfide bond between the two conserved cysteine residues (C199 and C208). In *Deinococcus radiodurans*, a previously unreported special characteristic of DrOxyR (DR0615) is found with only one conserved cysteine. *dr0615* gene mutant is hypersensitive to H_2_O_2_, but only a little to ionizing radiation. Site-directed mutagenesis and subsequent *in vivo* functional analyses revealed that the conserved cysteine (C210) is necessary for sensing H_2_O_2_, but its mutation did not alter the binding characteristics of OxyR on DNA. Under oxidant stress, DrOxyR is oxidized to sulfenic acid form, which can be reduced by reducing reagents. In addition, quantitative real-time PCR and global transcription profile results showed that OxyR is not only a transcriptional activator (e.g., *katE*, *drb0125*), but also a transcriptional repressor (e.g., *dps*, *mntH*). Because OxyR regulates Mn and Fe ion transporter genes, Mn/Fe ion ratio is changed in *dr0615* mutant, suggesting that the genes involved in Mn/Fe ion homeostasis, and the genes involved in antioxidant mechanism are highly cooperative under extremely oxidant stress. In conclusion, these findings expand the OxyR family, which could be divided into two classes: typical 2-Cys OxyR and 1-Cys OxyR.

## Introduction

Reactive oxygen species (ROS), including hydrogen peroxide (H_2_O_2_), superoxide, and hydroxyl radical, are toxic to cells due to their ability to damage DNA and especially proteins containing iron-sulfur clusters or sulfur atoms [1]. In bacteria, many transcription factors have been found to sense the presence of ROS and induce antioxidant system. OxyR is such a peroxide sensor and transcription regulator. It was originally identified in *Salmonella enterica* serovar Typhimurium and *Escherichia coli* [2], [3]. In *E. coli*, OxyR is a positive regulator of *dps* (a DNA-binding ferritin-like protein), *groA* (GSH), *grxA* (glutaredoxin), *katG* (catalase), *ahpCF* (alkylhydroperoxide-NADPH oxido-reductase subunits F and C), *fur* (Fe-homeostasis regulation), and *oxyS* (a regulatory RNA) [4]. However, OxyR acts as a repressor of catalase expression in *Neisseria gonorrhoeae* [5].

As a redox-responsive protein of the LysR family, OxyR has conserved regions consisting of a helix-turn-helix motif and a LysR-substrate binding domain. When the cells are exposed to H_2_O_2_, OxyR protein is thought to be activated via the formation of a disulfide bond between the two cysteine residues (C199 and C208) [2], [6]. Detailed footprinting studies indicate that oxidized OxyR binds to its target promoters as a tetramer, occupying four adjacent major grooves upstream of the genes to be transcriptionally activated [7]. However, Kim *et al*. argued that OxyR activation does not involve disulfide bond formation at all, and that only one thiol in OxyR is critical for protecting against H_2_O_2_ [8]. Their work disclosed that OxyR is not involved in disulfide bond formation when it was activated by S-nitrosylation, and that mutation of C208 (which was reported to form a disulfide bond with C199) would not result in the cell hypersensitivity to H_2_O_2_, whereas the mutation of C199 did [8].

The gram-positive bacterium *Deinococcus radiodurans* is well known for its extreme resistance to ionizing radiation [9], [10], ultraviolet radiation [11], [12], oxidizing agent [13], and desiccation [14]. It has been suggested that protective mechanisms against oxidative damage is also involved in this extreme radiation resistance [13], [15]. *D. radiodurans* possesses a powerful enzymatic antioxidant system, including three catalases, three superoxide dismutases, two Dps, etc. However, the mechanism of its response to oxidant stress has not been well clarified. Here, we demonstrate an OxyR in *D. radiodurans*, which is different from all reported homologs in containing only one cysteine residue. Based on quantitative real-time PCR (QRT-PCR), we found that DrOxyR is both an activator, and a repressor. The binding of purified His-tagged OxyR protein to the upstream region of the respective genes was verified *in vitro* by DNA band shift assays. Furthermore, we investigated the global trancriptome variation due to disruption of *droxyR*, and the comparative analysis reveals pathways significantly impacted either directly or indirectly by *droxyR*.

## Results

### Identification of oxyR in *D. radiodurans*

**Figure 1:**
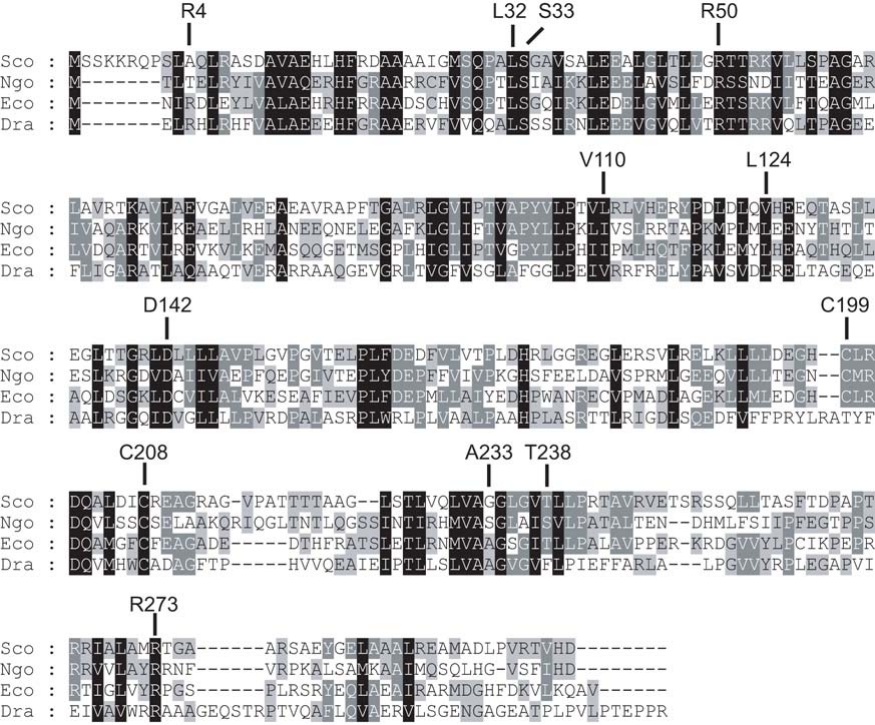
Alignment of OxyR homologs from different organisms. Using CLUSTAL W software aligned amino acid sequences of the *Streptomyces coelicolor* A3(2), *Neisseria gonorrhoeae*, *E. coli*, and *D. radiodurans*. Identical amino acids are highlighted in black, and conserved residues are highlighted with grey. The DrOxyR helix-turn-helix region has four conserved residues (R4, L32, S33 and R50) [17]. At its LysR-substrate binding domain, D142 and R273 possibly define an activating region on OxyR (contact with RNA polymerase)[18], A233 residue is involved in tetramerization[17], V110, L124, and A233 form a hydrophobic core[58]. Numbering is based on the *E. coli* OxyR sequence.

In *D. radiodurans* genome database (TIGRE), there is a potential *oxyR* homolog (DR0615, designated as *droxyR*) [15], which encodes a protein of 317 amino acids. BLASTP analysis showed that DR0615 exhibited 31% identity to *E. coli* OxyR and 29% identity to *N. gonorrhoeae* OxyR, respectively [16]. Five conserved residues in its helix-turn-helix region (between amino acids 3 to 62) involved in DNA binding are identical (Figure 1) [17]. Other functional domains are conserved at its LysR-substrate binding domain (between amino acids 86 to 297), including a hydrophobic core, a tetramerization domain, and a RNA polymerase binding domain [17], [18]. Interestingly, DR0615 has a single sensing cysteine residue (C210), compared with other organisms. This difference in the primary structure of *oxyR* raised the possibility that *droxyR* need not, indeed can not form an intramolecular disulfide bond, and that DrOxyR activation can be caused by the modification of just one cysteine residue (C_210_) (Figure 1).

### Phenotypic characterization of *MOxyR*

To test its role in the antioxidant mechanism of *D. radiodurans*, a *droxyR* disruptant strain (MOxyR) was constructed (Table 1). The coding region of the *dr0615* was replaced with a kanamycin resistance cassette under a constitutively expressed *D. radiodurans groEL* promoter. The primers are used for mutation listed in Table 2.

**Table 1:** Bacterial strains and plasmids used in this study

Stains and plasmids	Relevant genotype	Reference or source
***D. radiodurans***		
R1	ATCC 13939	[59]
MOxyR	*D. radiodurans* DR0615 gene knockout mutant	This work
MOxyR_wtC	MOxyR complement with pRAD*oxyR*	This work
MOxyR_sdC	MOxyR complement with pRAD*oxyR*sdC	This work
***E. coli***		
DH5α	Host for cloning vectors	Laboratory stock
E12	The parent strain of GS09, wild type strain	[7]
GS09	DH5α *oxyR* gene knockout mutant	[7]
GS09C	GS09 complement with pRAD*oxyR*	This work
BL21 (DE3)	*E. coli* B F^−^ *dcm ompT hsdS* (r_B_^−^m_B_^−^) *gal λ* (DE3)	Novagen
BLOxyR	BL21 containing expression plasmid pET28OxyR	This work
BLOxyRsd	BL21 containing expression plasmid pET28OxyRsd	This work
**Plasmids**		
pMD18	Cloning vector (Ap^r^)	Takara
pET28a	Expression vector (Km^r^)	Novagen
PMD18*oxyR*	*oxyR* gene is cloned to pMD18 (Ap^r^)	This work
pMD18*oxyR*sd	Site-directed mutant gene is cloned to pMD18 (Ap^r^)	This work
pRADK	pRADZ3 derivative in which lacZ is replaced by the kanamycin gene (Ap^r^ Km^r^ Cm^r^)	[43]
pRAD*oxyR*	pRADK derivative in which kanamycin gene is replaced by the *oxyR* gene from pMD18*oxyR* (Ap^r ^Cm^r^)	This work
pRAD*oxyR*sdC	pRADK derivative in which kanamycin gene is replaced by the *oxyR* site-directed mutant gene from pMD18*oxyR*sd (Ap^r ^Cm^r^)	This work
pET28*oxyR*	pET28a expression plasmid containing BamHI-NdeI fragment of oxyR from pMD18*oxyR* (Km^r^)	This work
pET28*oxyRsd*	pET28a expression plasmid containing BamHI-NdeI fragment of the site-directed mutant *oxyR* from pMD18*oxyR*sd (Km^r^)	This work

**Table 2:** Primers used in this study.

Primer	Sequence
Construction of *oxyR* mutants	
OxyR1	5′ TTGGCGAGATTGGGGTTGA 3′
OxyR2	5′ CACACA**GGATCC**GTACAGCTCCCGAAAGCG 3′
OxyR3	5′ AGAGTT**AAGCTT**CAGGAGGACTTCGTGTTTT 3′
OxyR4	5′ CCTCCCAAACGACAAATCCC 3′
OxyR5	5′ CCAGCGTGTCGTTGATGCG 3′
KanamycinF	5′ CACACAGGAAACAGCTATGACCATGATTA 3′
KanamycinR	5′ ACAGAC**GGATCC**TAGAAAAACTCATCGAGCATC 3′
Complementation of *oxyR* mutants	
OxyR_com_F	5′ TTT**CATATG**GAACTGCGACACCTGC 3′
OxyR_com_R	5′ TTT**GGATCC**ATGGTCATGGGAAAGCTCCTT 3′
Site-direct mutagenesis primer	
C_210_AF	5′ TTCGACCAGGTCATGCACTGGGCCGCCGACGCGGGCTTTACG 3′
C_210_AR	5′ CGTAAAGCCCGCGTCGGCGGCCCAGTGCATGACCTGGTCGAA 3′
Real-time quantitative PCR	
DR0089	F: 5′ GAAACAGGAGCGCAGGGTGT 3′
	R: 5′ AGGTTGCGTTGCAGGGTTTC 3′
DR0865	F: 5′ CCGGGGACATCATCACCATT 3′
	R: 5′ CCGGACACTGCCCATAAAGC 3′
DR1219	F: 5′ TACCTCGCCATGAGCTTTCT 3′
	R: 5′ CCCAGAATCAGCGGAATAAA 3′
DR1709	F: 5′ GCGATGGTGATTCAGAACCT 3′
	R: 5′ GTTCGGCCTGAATCCAGTAA 3′
DR1982	F: 5′ CACCAGCAGGCCGAGAAGTT 3′
	R: 5′ GTTGTCCTCACCGGGAATGC 3′
DR1998	F: 5′ GGGCGTGGACAAGCGTATTC 3′
	R: 5′ GTAGACGGGGGCTTCCTGCT 3′
DR2263	F: 5′ GAAACAGGAGCGCAGGGTGT 3′
	R: 5′ AGGTTGCGTTGCAGGGTTTC 3′
DRB0092	F: 5′ GCGACGTGGAAAAGGTGGAC 3′
	R: 5′ GCTTGCCGTTGTTGATGTCG 3′
DRB0125	F: 5′ GCACTGCCACTGTCAAGAAA 3′
	R: 5′ GTCCTGCGGCTCAAAGTAAG 3′
Gel mobility shift assays	
DR1709	F: 5′ GAGCCTCTAGCAAATATGTGACAGCAC 3′
	R: 5′ AGGCTGGGAGAACGGGAATC 3′
DR1998	F: 5′ GCGCAGGATAGCGGATGC 3′
	R: 5′ CCGACGCCCTTGTTGTTTTC 3′
DR2263	F: 5′ CTGGGCGCAGCTTGAAGTTA 3′
	R: 5′ CACGCCGCTCTTTTTGGTCT 3′
DRB0036	F: 5′ GAACACGATGTCCGCTGCAC 3′
	R: 5′ TTGAGGCCGTGCTGTCAGTC 3′
DRB0125	F: 5′ GCTGCTGGCTTTCCCTTCAG 3′
	R: 5′ GGGCGAGGGTCAGAAAAAGG 3′
DR0207	F: 5′ GGCTTGCAAGTGGAACCC 3′
	R: 5′ CAGCAGGGTCTTGGTCAT 3′
RV-M	5′ GAGCGGATAACAATTTCACACAGG 3′

**Figure 2:**
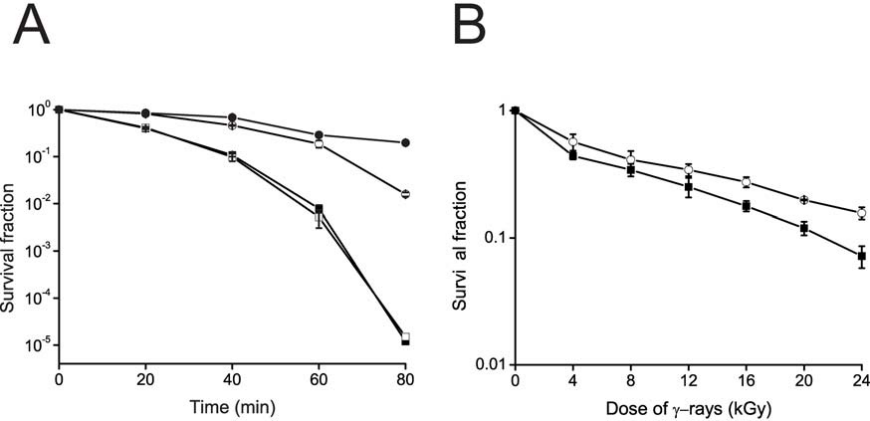
Survival curves of D. radiodurans strains exposed to (A) H_2_O_2_ and (B) ionizing irradiation. (A) Wild-type D. radiodurans R1 (○) compared to MOxyR (▪), MOxyR_wtC (•), and MOxyR_sdC (□) under 20 mM H_2_O_2_ treatment at the five recovery time points (0, 20, 40, 60, and 80 min). (B) Wild-type D. radiodurans R1 (○) compared to MOxyR (▪) after ionizing irradiation. Error bars represent standard deviations from four replicate experiments.

Disruption of *dr0615* did not show a growth defect (data not shown). However, as shown in Figure 2A, the sensitivity of the mutant after 20 mM H_2_O_2 _treatment was increased, compared to the wild type strain. After complemention with wild type *droxyR* gene (plasmid pRAD*oxyR*) (Table 1), H_2_O_2_ resistance of the MOxyR_wtC was significantly increased. In contrast, the MOxyR_sdC strains, which is the *oxyR* disruption mutant complemented with the *droxyR* C210A site-directed mutant (plasmid pRAD*oxyR*sdC), still showed sensitivity to H_2_O_2_. In addition, a little difference was observed between the ionizing radiation resistance of wild type strains and that of MOxyR (Figure 2B).

### Differences in catalase activities and ROS levels between the MOxyR and wild type strains

**Figure 3:**
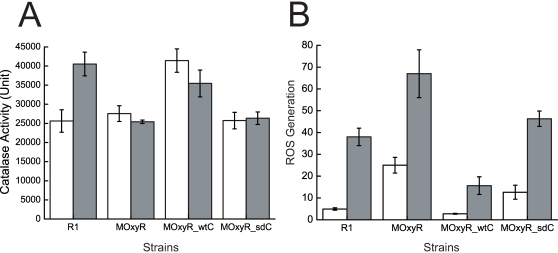
Effect of *oxyR* disruption on the antioxidant ability of *D. radiodurans*. (A) Catalase activities of R1, MOxyR, MOxyR_wtC, and MOxyR_sdC after H_2_O_2_ treatment (grey bar) or not (white bar). (B) ROS accumulate in four strains after H_2_O_2_ treatment (grey bar) or not (white bar). Data reported represent the average and standard deviations of three independent experiments.

In order to investigate the regulatory role of OxyR on enzymatic antioxidants of *D. radiodurans* after treatment of H_2_O_2_, we assayed the catalase activity in wild type, MOxyR, MOxyR_wtC, and MOxyR_sdC stains with H_2_O_2_ treatment or not (Table 1). When log-phase cells were treated with 20 mM H_2_O_2_, the wild type showed a 1.5-fold increase in catalase activity, whilst, neither MOxyR nor MOxyR_sdC showed an increase (Figure 3A). On the other hand, complement strain MOxyR_wtC showed a higher catalase activity than the wild-type under normal conditions. It is well accepted that *oxyR* expression is auto-regulated via negative feedback in *E.coli* [19], so we presume that the *droxyR* gene is under the control of the stronger *groEL* promoter in pRAD*oxyR*, the transcription of which destroys the negative feedback. As a result, catalase production may have been activated by an abundance of OxyR, which is likely oxidized by H_2_O_2_ from normal metabolism. This might be the reason why MOxyRC is more resistant to hydrogen peroxide than the wild type strain. These results indicate the *droxyR* gene is responsible for the regulation of catalase activity.

To determine whether the *droxyR* disruption has effect on the total ROS scavenging ability of the cell, we also measured the intracellular ROS level. Figure 3B shows that ROS level in all the strains increased after the H_2_O_2_ treatment, and that MOxyR and MOxyR_sdC accumulated more ROS than wild type and MOxyR_wtC.

Combined with the survival phenotypic data, it could be inferred that the sensitivity of mutant to H_2_O_2 _is due to the loss of induction effect of *oxyR* on antioxidant enzymes (e.g. catalase), and that *oxyR* acts as a positive regulator of catalase. Moreover, the C210A mutant showed the same phenotype as MOxyR, indicating that C210 is a site key to OxyR gene regulation.

### C210 is a sensing cysteine

**Figure 4:**
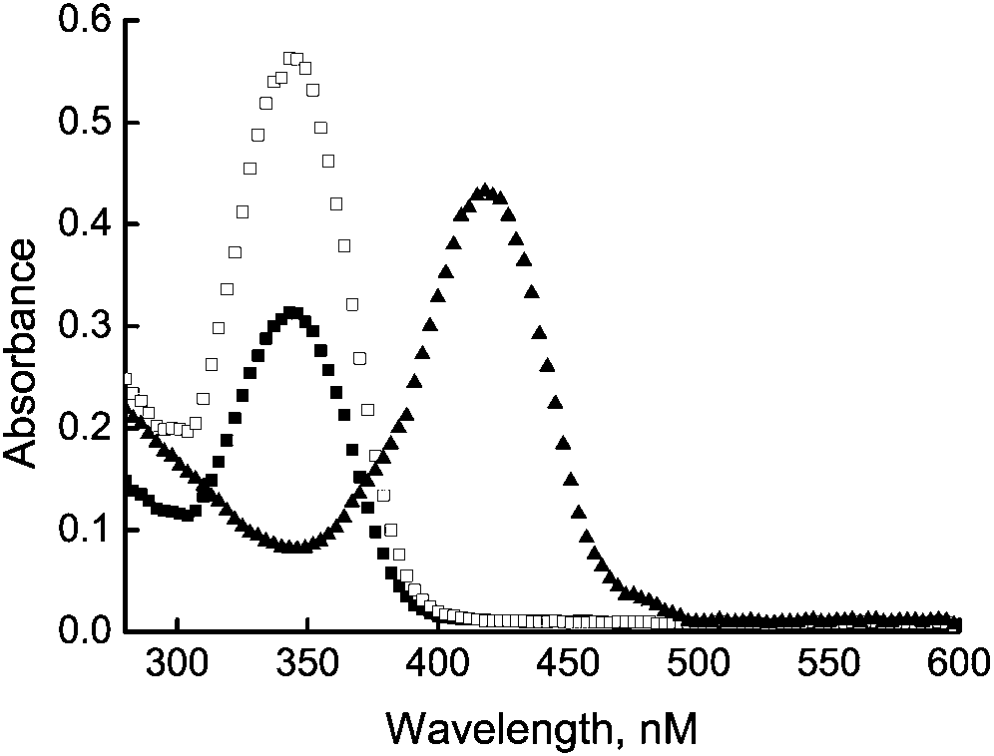
Absorbance of NBD chloride-treated purified OxyR. Air-oxidized OxyR (▪), along with the CHP (10 mM) treated OxyR (□), shows maximal absorption at 347 nm. The shoulder at 420 nm is reduced OxyR (▴) reacted with NBD chloride. The absorbance spectrum is from 285 to 600 nm.

The site-directed mutagenesis of *droxyR* revealed that residue C210 plays essential roles in the function of the protein. This finding poses an intriguing question that whether C210 is a sensing cysteine. To verify this hypothesis, experiments were carried out to obtain C_210_-SOH formation by either CHP or air oxidation, followed by the use of an electrophile (NBD-Cl) to trap the C-SOH [20]. As expected, OxyR treated with CHP (Cys-210SO-NBD) exhibited a maximal absorbance at 347 nm (Figure 4), on the hand, the reducing form Cys-210S-NBD showed its maximal absorbance at 420 nm. This data identified that C210 is the peroxidatic center of the molecular.

Because sulfenic acid is highly unstable, and reacts further to produce a disulfide, we used nonreducing SDS-PAGE to identity whether C210 is involved in intermolecular disulfide bond formation. As typical OxyR protein, DrOxyR protein did not form intermolecular disulfide linkages after CHP (organic hydroperoxide) or H_2_O_2 _(inorganic hydroperoxide) treatment (data not shown).

### QRT-PCR analysis disclose two classes of OxyR-dependent genes

As in the *oxyR* knockout of *E. coli*., disruption of *oxyR* makes *D. radiodurans* much more sensitive to H_2_O_2_. As we have known, this sensitivity is attributed to the inhibition of normal transcription of OxyR-dependent genes [16], [21]–[23]. Therefore, eight homolog transcripts which were reported as OxyR-dependent genes in other bacteria [21], [23]–[25] were selected and compared in wild type and MOxyR using QRT-PCR. QRT-PCR was also used to analyze the expression patterns of these genes in wild type and MOxyR after treatment with H_2_O_2_. Of these genes, *dr1998* codes a major catalase KatE in *D. radiodurans*, and it has been shown to play a role in protection of *D. radiodurans* from oxidative stress and ionizing radiation [13]. *dr2263* and *drB0092* are two *dps* genes, with functions of protection against oxidative stress and iron uptake and storage [26], [27], but their expression patterns are different under ionizing radiation [28], indicating that their regulator may be different. Furthermore, *D. radiodurans* accumulates high intracellular manganese and low iron levels compared with radiation-sensitive bacteria and this is regarded as an important contribution to its resistance [29]. Three ion transport genes were selected to test whether they are regulated by OxyR, including *dr1219* (*feoB,* coding ferrous iron transport protein B), *drB0125* (coding Iron(III) dicitrate-binding periplasmic protein), and *dr1709* (*mntH*). In addition, DR1982 is an alkylhydroperoxide reductase subunits F, which could transfers electrons from NADH to AhpC. DR0865 is a Fur or PerR homolog, and Fur proteins control iron uptake in many Gram-negative bacteria, while PerR is postulated to be the peroxide regulon repressor [30].

**Figure 5:**
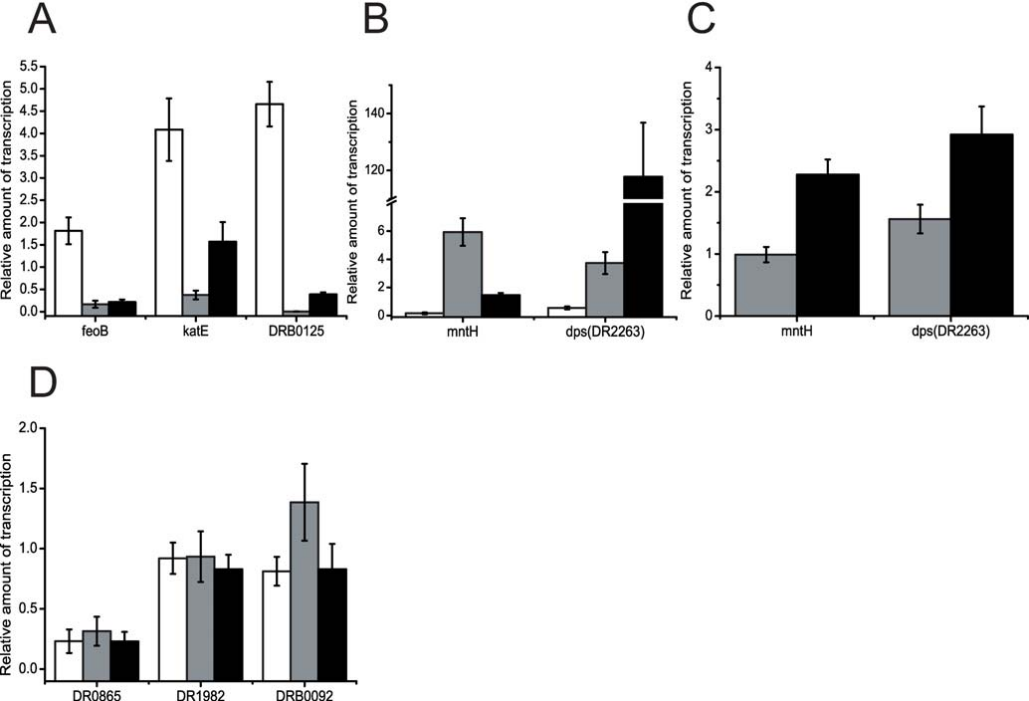
Q-RT PCR of the expression of potential OxyR-dependent genes in *D. radiodurans* R1 wild-type compared to wild type after H_2_O_2_ treatment (white bar), MOxyR (grey bar), and MOxyR H_2_O_2_ treatment (black bar). (A) genes positively regulated by DrOxyR; (B) genes negatively regulated by DrOxyR; (C) the expression patterns of DR1709 and DR2263 were measured in MOxyR_sdC; (D) genes not regulated by DrOxyR.

Consistent with catalase activity assay, the *katE* (*dr1998*) transcript was repressed approximately 1.74-fold in the MOxyR relative to that of wild type, and induction of *katE* expression by H_2_O_2_ was eliminated in strain MOxyR (Figure 5A), suggesting that OxyR is a positive regulator of *katE*. In addition, both iron transporter genes (*dr1219* and *drB0125*) showed the same expression pattern as *katE*, indicating that expression of these genes were also mediated by OxyR, a finding similar to the OxyR regulon in *Haemophilus influenzae* [23].

When the wild type cells were exposed to H_2_O_2_, the transcripts of *dps* (*dr2263*) and *mntH* (*dr1709*) decreased, whereas, transcripts of these genes in the MOxyR cell were identified either under normal growth conditions or after H_2_O_2 _challenge (Figure 5B). These data informed us that oxidized DrOxyR might act as a transcription repressor of *dr2263* and *dr1709*. Since the expression levels of *dr2263* and *dr1709* are higher in MOxyR than those of in wild type strains under normal condition, we deduce that reduced DrOxyR could also be a repressor of both of them. To verify this hypothesis, we measured the expression patterns of DR1709 and DR2263 in MOxyR_sdC, which contained a reduced OxyR protein due to its C210 mutation. Opposite to their expression patterns in MOxyR, both of them did not show a significant increase under normal condition (Figure 5C). Although they were activated under H_2_O_2_ treatment in MOxyR_sdC, the change fold is less than that of in MOxyR. The data confirmed that reduced OxyR could still negatively regulate these genes expression. DrOxyR action pattern is opposite to its homologs in *E. coli* [21], *Bacteroides fragilis* [31], and *Shigella flexneri* [32]. In *D. radiodurans*, two types of Mn(II) import systems have been identified, DR1709 belongs to the Nramp family of transporters [29]. Another type of predicted Mn transporter is an ATP-dependent ABC-type transporter (DR2283-DR2284, DR2523) [29]. However, QRT-PCR results showed that the second Mn ion transporter system was not regulated by DrOxyR protein (Data not shown).

Additionally, *dr1982* (*ahpF*) and *drB0092* (*dps*) did not show significant changes at oxidant stress (Figure 5D). We deduced that OxyR did not regulate both of them, and the transcription patterns of the two *dps* genes (*dr2263* and *drB0092*) are different. Furthermore, we also measured the expression of *dr0865*, which is a putative *perR* homolog. We found that *katE* was significantly activated after deletion of *dr0865*, and that the mutant strain exhibited greater resistance to H_2_O_2_ than wild type strain (unpublished data). Interestingly, the expression level of *perR* was repressed in MOxyR, as well as in the wild type strain after H_2_O_2_ challenge. Given the expression pattern of *perR*, the oxidant stress occurred after disruption of *oxyR*, which was consistent with intracellular ROS accumulation assay results.

Since the Mn(II) transporter gene (*dr1709*) is induced, and the iron transporter genes (*dr1219*, *drB0125*) are repressed in *oxyR* mutant, we assayed the intracellular Fe ion and Mn ion levels in MOxyR. As expected, compared to that Fe ion levels are three times higher than Mn ion in wild type strain, Fe ion levels are only two times higher than Mn ion in MOxyR (Table 3).

**Table 3:** Intracelluar Mn and Fe levels in wild type and MOxyR.

Strains	Total Mn (nmol Mn/mg cell)	Total Fe (nmol Fe/mg cell)	Intracelluar Mn/Fe concentration ratio
Wild type	1.017±0.24	3.166±0.54	0.321
MOxyR	1.517±0.31	3.046±0.48	0.498

### Transcriptome changes in response to disruption of *droxyR*

The results of intracellular ROS accumulation assay showed that MOxyR accumulates higher levels of ROS than the wild type strain. It is well known that ROS is a signaling molecule, and has an important role in the regulation of a variety of biological processes [33], [34]. Therefore, in order to identify other OxyR-dependent genes, and to measure the consequences of higher levels of ROS, we carried out a microarray comparison of the wild type and the OxyR mutants grown under normal conditions. Table S1 and Table S2 exhibits that a total of 280 genes showed at least a 2-fold change (*p*<0.05). A higher percentage of genes were repressed (150 genes, Table S1), whereas, 130 genes were induced (Table S2). Table 4 and Table 5 showed the 36 most highly repressed and induced genes, many with known roles in oxidative stress response, including catalase, oxidoreductase, N-acetyltransferase. Furthermore, functional classification of these genes revealed that signal transduction mechanisms, inorganic ion transport and metabolism, lipid metabolism, energy production and conversion, and amino acid transport and metabolism showed altered expression patterns in the *oxyR* mutant (Table S3).

**Table 4:** The 36 most highly repressed genes in MOxyR.

Locus	Annotation	Repression fold	*P* value
DR0096	ABC Transporter with ATPase domain	34.94564	2.68E-05
DR1440	Cation-transporting ATPase, authentic frameshift	26.7681	0.000119
DRA0157	Periplasmic phosphate-binding protein PSTS	22.29369	0.000648
DR2209	Rio1 family Protein kinase	21.49044	5.80E-05
DR1263	YBIA_ECOLI in E coli	20.82255	0.001641
DRB0067	Extracellular nuclease with Fibronectin III domains	12.38872	4.75E-06
DR0095	ABC Transporter with ATPase domain	11.56158	7.25E-05
DRA0019	N-acetyltransferase	9.147487	0.002963
DRA0159	Phosphate transport permease PSTA	8.3616	2.35E-05
DR1624	RNA helicase, authentic frameshift	8.004592	2.01E-05
DR2409	McrA family nuclease	6.317552	0.00069
DR0548	Pilin IV like secreted protein	6.205551	1.20E-06
DR0353	VacB, S1 domain nuclease	6.171572	5.28E-05
DR1978	N-acetyltransferase family	6.035044	0.003969
DR1998	Catalase, CATX	6.024595	0.000272
DRA0020	COBU cobinamide kinase/cobinamide phosphate guanylyltransferase	5.547864	0.00179
DRA0017	Thioesterase	5.391183	0.00152
DR2449	Small conserved bacterial proteins, YOHL_ECOLI	5.184686	0.00625
DR2339	LigT, 2′-5′ RNA LIGASE	5.172343	0.001245
DR0352	Predicted protein	4.952399	0.000498
DR2441	N-acetyltransferase	4.836622	0.000183
DR0396	Predicted protein	4.642973	0.000548
DR2453	P-type ATPase metal efflux	4.543941	0.00816
DR0533	Predicted protein	4.380241	0.001366
DR1630	Conserved transport protein	4.305581	0.012968
DR1583	Conserved membrane protein	4.254401	0.000343
DR1200	Predicted protein	4.061794	0.003261
DR2385	Phenylacetic acid degradation protein PaaB	4.044868	0.000759
DR0746	Predicted protein	4.032291	0.00016
DR2208	Glyoxalase/Dioxygenase family protein	3.964627	0.01098
DR1976	MutS- Mismatch repair, ABC superfamily ATPase	3.93664	0.00046
DR1419	P-loop containing protein, tunicamycin resistance protein ortholog	3.903037	0.011378
DR2384	Phenylacetic acid degradation protein PaaC	3.882149	3.92E-05
DR1582	Conserved membrane protein	3.770361	0.000821
DR0203	Membrane protein, similar to gi|1653436 of Synechocystis	3.729993	0.022306
DR1907	Fe-S subunit of glycolate oxidase, YKGE	3.728085	0.007343

**Table 5:** The 36 most highly induced genes in MOxyR.

Locus	Annotation	Induction fold	*p* value
DRA0211	HTH transcriptional regulator, GntR family	12.78088	0.001481483
DR0959	ABC transporter permease dipeptide transporter	12.25984	6.45E-05
DRA0154	Glutamine-fructose-6-phosphate transaminase, GLMS	11.46216	0.001714972
DR1930	Membrane protein	8.843763	0.002494076
DR1317	Predicted protein	7.865743	0.000207444
DR1312	Nuclease McrA superfamily	7.19721	0.003461522
DRA0343	Succinic semialdehyde dehydrogenase	7.002605	0.001362937
DR2576	DHH family phosphohydrolase	6.333434	0.004310024
DR1790	Yellow/royal jelly protein of insects	6.152674	0.000235587
DR1901	Predicted protein	6.112743	0.00459121
DR1225	Glycosyltransferase	5.776983	0.005089054
DR2006	Predicted protein	5.489444	0.005599313
DRB0017	Vibriobactin utilization protein viub	5.484233	0.005609405
DR1385	Predicted protein	5.327047	0.001640339
DR1809	Glycine dehydrogenase, glycine cleavage system P protein	5.307754	6.59E-05
DR2181	RAB/RAS like small bacterial GTPase	5.306127	0.000119887
DR1231	Predicted protein	5.285835	0.001626178
DR1811	Glycine cleavage system H protein	5.047402	0.001147252
DR2179	Predicted protein	4.948525	3.11E-05
DRA0064	Thermostable? alkaline proteinase	4.907261	0.006968747
DRA0245	Predicted protein	4.604159	0.007938807
DR0645	Molybdopterin-guanine dinucleotide biosynthesis protein A	4.529622	0.008214151
DRA0192	Predicted protein	4.416647	0.008664458
DRA0333	Zn-finger+FHA domain containing protein	4.284262	0.000166068
DRB0036	Oxidoreductase, authentic point mutation	4.163247	0.015962058
DRA0230	Predicted protein	4.049166	0.000261909
DRA0331	VWFA Mg-binding domain protein	3.974087	0.000165997
DR0995	Protein of uncharacterized conserved family	3.861446	0.008895311
DR2182	Predicted protein	3.851292	0.003993378
DR2180	Uncharacterized small family of predominantly archaeal proteins	3.786965	0.00011024
DR2249	Protein phosphatase, calcineurin like phosphoesterase	3.756406	0.012430329
DRA0334	PP2C phosphoprotein phosphatase	3.671103	0.000141014
DR1623	Predicted protein	3.655162	0.016431343
DR1620	Oxidoreductase	3.649256	0.00096162
DR0751	Cell division topological specificity factor, MinE	3.571127	0.000262718

Table 6 and Table 7 show genes with the same expression pattern in wild type strain after 20 mM H_2_O_2_ treatment (our unpublished data) and these in MOxyR. This confirmed that oxidative stress occurs in MOxyR.

**Table 6:** Genes with a decreased level of expression both in wild-type strains treated with H_2_O_2_ (20 mM) and in MOxyR.

Locus	Annotation	MOxyR		H2O2 a	
		Decrease fold	*p* value	Decrease fold	*p* value
DR0019	FTSZ fragment	3.15	0.004408	2.92	0.062473
DR1198	TYPA like GTPase	2.02	0.022671	3.87	0.011932
DR1200	Predicted protein	4.06	0.003261	3.67	0.02233
DR1263	YBIA_ECOLI in E coli	20.82	0.001641	7.19	0.001764
DR1799	Initiation factor IF-2	2.53	0.002127	2.36	0.069017
DR1907	Fe-S subunit of glycolate oxidase, YKGE	3.73	0.007343	5.66	0.003095
DR2470	Related to biothin biosysnthesis protein BioY	2.51	0.027048	2.62	0.029802
DR2524	Ribosomal protein L28	2.47	0.005166	2.11	0.01617
DRA0157	Periplasmic phosphate-binding protein PSTS	22.29	0.000648	15.00	0.004186
DRA0158	Phosphate transport system permease PSTA	3.53	0.006286	2.74	0.041144
DRA0159	Phosphate transport permease PSTA	8.36	2.35E-05	2.72	0.031496
DRB0067	Extracellular nuclease with Fibronectin III domains	12.39	4.75E-06	72.34	5.90E-05
DRB0106	Acyl-CoA Thioesterase superfamily protein	3.20	0.000486	11.17	0.000729
DRB0107	NRDI ribonucleoprotein	2.34	0.002171	4.49	0.012634
DRB0111	Glycerophosphodiester phosphodiesterase	2.30	0.009128	2.95	0.017399

a These data are our unpublished data.

**Table 7:** Genes with an increased level of expression both in wild-type strains treated with H_2_O_2_ (20 mM) an in MOxyR.

Locus	Annotation	MOxyR		H2O2 a	
		Increase fold	*p* value	Increase fold	*p* value
DR0201	Predicted protein	2.92	0.00141	2.56	0.006516
DR0371	Predicted protein	2.57	0.007263	3.19	0.090488
DR0407	Membrane protein	2.65	0.030893	2.05	0.105049
DR0685	Uncharacterized secreted protein	2.15	0.000489	4.17	0.01594
DR0781	CHEY family+HTH domain	2.18	0.000696	2.05	0.010041
DR0894	Predicted protein	3.41	0.002955	3.33	0.007428
DR0959	ABC transporter	12.26	6.45E-05	2.37	0.015601
DR1179	HKD superfamily hydrolase	3.21	0.000231	2.95	0.005812
DR1306	Predicted protein	2.27	0.007313	2.75	0.045296
DR1314	Uncharacterized proteins, ysnf-like repeats	2.68	0.000105	2.20	0.06613
DR1331	Predicted protein	2.31	0.002401	2.69	0.03801
DR1385	Predicted protein	5.33	0.00164	2.50	0.029004
DR1697	Predicted protein	2.81	0.006795	4.11	0.006687
DR1708	Predicted protein	2.86	3.49E-05	2.58	0.025686
DR1803	Predicted protein	2.49	0.048071	23.78	0.000232
DR1804	Solo Double stranded beta helix protein	2.63	0.000896	2.59	0.017027
DR1811	Glycine cleavage system H protein	5.05	0.001147	2.18	0.326216
DR1879	Conserved membrane protein	2.35	0.005824	2.18	0.087706
DR1901	Predicted protein	6.11	0.004591	2.01	0.111755
DR1980	Rossman fold oxidoreductase	2.14	0.00147	2.17	0.033191
DR1987	Predicted protein	2.62	0.000202	2.51	0.019178
DR2179	Predicted protein	4.95	3.11E-05	2.32	0.064013
DR2235	PHP family phosphoesterase	2.01	0.033357	2.04	0.092577
DR2240	Predicted protein	2.82	0.001259	2.89	0.031108
DR2438	Endonuclease III	2.10	0.002349	3.69	0.01421
DR2527	Predicted protein	2.19	0.002913	2.08	0.038997
DRA0005	NAD alcohol dehydrogenase	2.45	0.039132	9.12	0.004303
DRA0334	PP2C phosphoprotein	3.67	0.000141	2.20	0.059918
DRA0343	Succinic semialdehyde dehydrogenase	7.00	0.001363	2.24	0.230105
DRA0364	ADG Oxidoreductase	2.48	0.001306	4.51	0.008529

a These data are our unpublished data.

### Gel mobility shift assays to confirm OxyR-regulated genes

**Figure 6:**
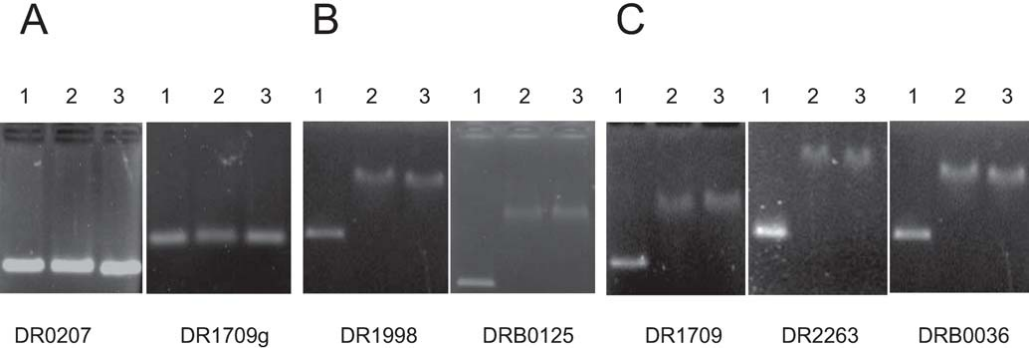
Binding of reduced and oxidized OxyR to the upstream region of (A) negative control (*dr0207* and coding sequence for *dr1709*); (B) positivly regulated genes (*dr1998* and *drB0125*); (C) negativly regulated genes (*dr1709*, *dr2263*, and *drB0036*). To generate reduced protein, 200 mM DTT was added to the binding reactions. Column 1, 2, and 3 indicate non-added protein, reduced OxyR added, and oxidized OxyR added, respectively.

Our global transcriptome analysis results suggested that the expression patterns of many genes were altered as a consequence of *oxyR* deletion. However, microarray data could not distinguish those genes directly controlled by OxyR form those controlled by indirect mechanisms. To support the reliability of both QRT-PCR and microarray data, we used a DNA mobility shift assay to determine whether purified OxyR protein could bind *in vitro* to the two potentially positively regulated gene (*dr1998* and *drB0125*) and two potentially negatively regulated genes (*dr1709* and *dr2263*). In addition, microarray data showed that many oxidoreductase genes were repressed, so we cloned the promoter sequence of *drB0036* which is induced after ionizing radiation [28] to identify whether *oxyR* is a regulator of oxidoreductase. *dr0207* was used as a negative control, which is up-regulated after ionizing radiation[35]. As shown in Figure 6, both oxidized and reduced forms of the protein could bind these promoters. Given that OxyR was not completely reduced with DTT and the DTT was probably quickly electrophoresed away from the protein in the mobility shift assays [7], we also examined the binding of the C210A mutant protein to these genes and observed a same binding pattern (data not shown). This data indicate that OxyR protein is bound to its recognition sequences even in the absence of oxidative stress, and the binding ability supports the result that reduced OxyR could regulate *dr1709* and *dr2263* expression.

## Discussion

*D. radiodurans* exhibits extreme radiation resistance. In addition to its powerful DNA repair systems which include some novel components [36], free radical scavengers are regarded as important contributors to this resistant mechanism [15], [37]. Recently, Daly *et al.* reported that accumulation of Mn (II) in *D. radiodurans* facilitates its radiation resistance through high levels of protein protection from oxidation [29], [38]. However, despite these efforts, the molecular regulatory mechanism underlying its oxidative resistance remains poorly understood. Therefore, in this work, we demonstrate that DR0615 is an OxyR homologue with an important role in oxidative stress sensing and regulation mechanism.

Unlike many other OxyR homologs, the sequence analyses showed that DrOxyR contains only one cysteine, which is absolutely conserved in other OxyR homologs (Figure 1). Further comparisons of the amino acid sequences of DrOxyR with those from other bacteria indicated that the other activating regions are well conserved in the DrOxyR protein, except the T238 residue which is involved in C199–C208 disulfide bond formation is absent (Figure 1) [18]. This sequence characteristic informed that DrOxyR could not form intramolecular disulfide bond under oxidative stress. However, disulfide bonds are not the only cysteine oxidation product important for redox sensing. As *Bacillus subtilis's* OhrR protein [39], our NBD chloride assay *in vitro* showed that the sole cysteine could be oxidized into sulfenic acid and did not generate an intermolecular disulfide bond. The *in vivo* functional analyses of the cysteine mutant and wild type OxyR showed that the single C210 is sufficient for DrOxyR to act as a sensor of and a regulator responding to oxidant stress. But the protein may react with low molecular weight thiols (e.g. Cysteine) to make a mixed disulfide *in vivo,* such as that of BsOhrR [40]. This is a major mechanistic difference from the sensing mechanism of the 2-Cys OxyR, for which sulfenic acid is an intermediate in the pathway to intramolecular disulfide bond formation [2]. Nevertheless, this activating pathway was challenged by the report of Stamler's group, whose research showed that sulfenic acid is stable in per monomer [8].

Furthermore, the *droxyR* could not complement the defect in the *E. coli oxyR* mutant strain (GS09) (Figure S1), even when GS09 was complemented with *droxyR*::T201C, which is put the missing cysteine back in DrOxyR (data not shown). One explanation for the inability of DrOxyR to complement to GS09 is an inability to properly contact or communicate with *E. coli* RNA polymerase. Another explanation is that these two families (based on the number of cysteine residues present) use different mechanisms to activate downstream process.

Based on QRT-PCR results, eight potential OxyR-regulated genes were classified into three classes due to their different expression pattern in wild type and mutant strains before or after H_2_O_2 _stress. Particularly interesting is, excepting as a regulator of *katE*, DrOxyR also acts as an activator of iron transport genes and as a repressor of manganese transport gene. As seen in recent studies, the high intracellular Mn/Fe ratio in *D. radiodurans* plays an important role in resistance ability by protecting cells from ROS generation during recovery [15], [29], [38]. In addition, our findings do not preclude the existence of other regulators of Mn/Fe transport genes, or *katE*. Indeed, *dr0865* (*fur or perR*) and *dr2519* (*mntR* or *dtxR*) also showed the abilities to regulate these genes (our unpublished data), indicating that the oxidative stress response network is much more complex than we initial prediction.

From the microarray data, we found a total of 280 genes (about 9% of genome) that showed at least 2-fold change, suggesting that these genes were regulated by *droxyR* through either direct or indirect mechanisms. Several genes annotated as *N*-acetyltransferase (*dr0763*, *dr1057*, *dr1978*, *dr2441*, and *drA0019*) were repressed. In *Saccharomyces cerevisiae*, *N*-acetyltransferase could reduce intracellular oxidant levels and protect cells from oxidative stress [41]. Furthermore, genes involved in electron transport were also repressed, including *dr0343*, *dr1493*, *dr1502*, *dr1505*, and *dr2618*. This may result in a decrease of the production of ROS generated from the electron transport process [1], [15]. In addition, an iron-sulfur protein (DR1907) was also significantly inhibited to avoid the attack by ROS. Conversely, many oxidoreductases were overexpressed, some of which are involved in regulating the oxidation state and activities of several proteins [34]. Thioredoxin (DRA0164) is such an oxidoreductase whose expression level was highly elevated in MOxyR. It directly regulates the activation of specific signal transduction proteins through hydrogen peroxide-sensitive noncovalent interactions [34]. Moreover, gel mobility shift assays showed that DRB0036 (oxidoreductase) is under the control of OxyR, suggesting that OxyR may be directly involved in the oxidoreductase processes of *D. radiodurans*.

As a signalling molecule, hydrogen peroxide has an important roles in the regulation of a variety of biological processes, such as stimulate cell proliferation [33], [34]. In this work, some genes involved in transcriptional regulation, transport and cell proliferation also showed an altered expression pattern. Thus, it was deduced that these genes were not regulated by *oxyR*, but were affected as a consequence of ROS accumulation. For example, we have shown the expression levels of two genes, *minE* (*dr0751*) and *minD* (*dr2383*), were changed. The MinE protein, which is known to prevent the division inhibitor from acting at internal division sites, was activated, whereas the *minD* gene, which is a cell division inhibitor, was repressed. Based on these expression patterns, it could be assumed that the cell was stimulated due to ROS accumulation. Other interesting genes that were down-regulated in MOxyR were some DNA damage response genes, including *recA*, *cinA*, *ligT*, *dinB*, *ddrB*, *ddrC*, *ddrH*, *ddrJ*, *ddrK*, *ddrM*, and *ddrO* [14], whereas they were up-regulated in MOxyR after 20 mM H_2_O_2_ treatment (data not shown), indicating that they were not regulated by *oxyR* only.

**Figure 7:**
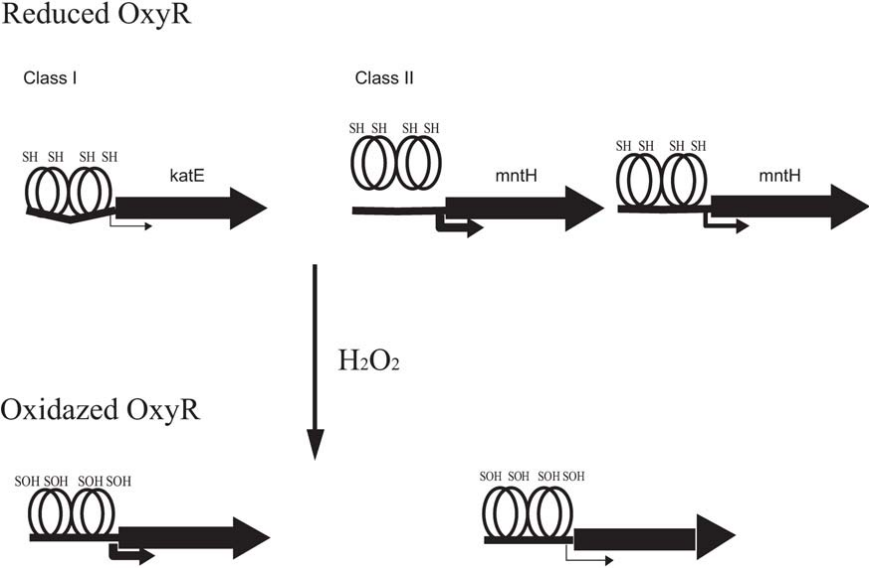
Model for reduced and oxidized OxyR binding to and activation at the two classes genes. For Class I (*katE*): OxyR activates gene in the presence of H_2_O_2_, whereas under non-stressed conditions, reduced OxyR is bound to two pairs of adjacent major grooves separated by one helical turn of the DNA duplex and acts to repress its own synthesis. For Class II (*mntH*): mutation of OxyR can greatly enhance gene expression, reduced OxyR binds to DNA and minimally induced genes, whereas oxidized OxyR significantly decreases the gene expression levels.

Based on the gene expression patterns, two classes of OxyR-dependent genes were identified and DrOxyR can function not only as a transcriptional activator but also as a transcriptional repressor. Our DNA band shift assay showed that either reduced OxyR protein or oxidized protein can bind both classes of genes. As transcriptional activator, reduced OxyR binds to two pairs of adjacent major grooves separated by one helical turn of the DNA duplex and acts to repress its own synthesis. When oxidized, the OxyR tetramer binds four adjacent major grooves upstream of those genes that are transcriptionally activated by OxyR [4], [7] (Figure 7). Whereas a repressor, the reduced form of OxyR produces a basal expression level in the absence of exogenous H_2_O_2_ and the oxidized form of OxyR significantly repressed the genes' expression level. Moreover, after deletion of OxyR, the expression of these genes was significantly induced in MOxyR (Figure 7). This agrees with published data showing that oxidized OxyR also acts as a repressor [5], [42]. Nonetheless, no obvious binding sites, such as those identified in *E. coli*, were observed in these genes. This is consistent with the results of OxyR-binding sites of genes in other bacteria [5], [22].

In summary, our work presents a biochemical mechanism for hydrogen peroxide sensing of *D. radiodurans* OxyR, which contains only one conserved cysteine. The gene transcription induction by hydrogen peroxide requires only one cysteine that can be reversibly oxidized by peroxides to a sulfenic acid form. Moreover, based on QRT-PCR and globe transcriptome analysis, we provide evidence that DrOxyR functions as not only a positive regulator but also as a negative regulator of different classes of genes. These results show that genes participating in the Mn/Fe homeostatic and antioxidant system are highly cooperative under extreme conditions, and that cooperation contributes to resistance. More research is needed to establish the detailed mechanism of OxyR regulation of these important genes, and whether communication between OxyR and other regulators such as PerR (DR0865) existed and is required for the intricate coordination of oxygen radical detoxification.

## Materials and Methods

### Strains and growth conditions

Bacterial strains and plasmids are listed in Table 1. All *D. radiodurans* strains used in this work were grown at 30°C in TGY (0.5%Tryptone, 0.3% yeast extract, 0.1% glucose) broth or on TGY plates supplemented with 1.5% Bacto-agar. Overnight cultures were incubated into fresh TGY medium and exponential-phase cells were used in all experiments. *E. coli* strains were grown in Luria-Bertani (LB) broth or on LB agar plates at 37°C.

### Disruption of the *dr0615* locus in D. radiodurans

A three-step gene splicing by overlap extension was used to generate the DR0615 mutant strain (designed MOxyR) [43]. Primers OxyR1 and OxyR2 were used to amplify a *Bam*HI fragment upstream of targeted genes, and primers OxyR3 and OxyR4 were used to obtain a *Hin*dIII fragment downstream of targeted genes respectively (Table 2). The kanamycin resistance cassette containing the *groEL* promoter was obtained from a shuttle plasmid, pRADK [43]. After these three DNA fragments were digested and ligated, the ligation products were used as template for PCR to amplify the resulting PCR fragment (OxyR1 and OxyR5 used as primer), which was then transformed into exponential phase cells by CaCl_2_ treatment. The mutant strains were selected on TGY agar plates supplemented with 20 µg/mL kanamycin were confirmed by PCR product sizes, enzyme-digested electrophoresis (Figure S2), and DNA sequencing.

### Complementation of *oxyR* mutant

Complementation plasmid construction was constructed as previously described by Hua *et al* [44]. Briefly, chromosomal DNA was isolated from wild type strains. A 1000-bp region containing the *oxyR* gene was amplified by OxyR_com_F and OxyR_com_R (Table 2), and ligated to pMD18 T-Easy vector (Takara, JP), designed as pMD*oxyR*. After digested by *Nde*I and *Bam*HI, the target gene *oxyR* was ligated to *Nde*I and *Bam*HI-pre-digested pRADK [43], which named as pRAD*oxyR*. The complementation plasmids were confirmed by PCR and DNA sequence analysis, and transformed into MOxyR and GS09 (K12 *oxyR*::*Kan* of *E. coli*, a gift from Dr Gisela Storz) [7], resulting in two functional complementation strains: MOxyR_wtC (*D. radiodurans oxyR* mutant strain complemented with pRAD*oxyR*) and GS09C (GS09 complemented with pRAD*oxyR*) (Table 1).

### PCR mutagenesis C_210_ of OxyR

The first PCR fragment was obtained using primer OxyR_com_F and a mutagenic antisense primer C_210_AR (Table 2, the mutated bases are underlined). The second PCR fragment was obtained using primer OxyR_com_R and the mutagenic sense primer C_210_AF, which is complementary to the C_210_AR. The mutagenesis PCR was generated by using OxyR_com_F and OxyR_com_R [39], and ligated to pMD18 T-Easy vector (Takara), designed as pMD18*oxyR*sd. Then, pMD18*oxyR*sd was digested with *Nde*I and *Bam*HI, and cloned into pRADK. The resulted pRAD*oxyR*sdC plasmid was transformed into DrOxyR. The *oxyR* site-directed mutation sequence was verified by sequencing.

### Measurement of cell survival rate

The sensitivity of *D. radiodurans* cells to hydrogen peroxide was assayed following the method as previously described with some modifications [45]. Cells were harvested in early stationary phase, washed twice with and re-suspended in phosphate buffer (20mM, pH 7.4). An aliquot was removed as control and the remaining aliquot was treated with hydrogen peroxide to a final concentration of 20 mM. The mixture was incubated at 30°C in an orbital shaker. At the indicated recovery time points (20, 40, 60, and 80 min), an aliquot was removed and catalase (Sigma, St. Louis, MO) was added in excess (100 g/mL) to stop the H_2_O_2 _treatment. The cells were diluted and spread on solid TGY media for determining the numbers of colonies forming units (cfu). Survival rates are defined as a percentage of the number of colonies obtained comparing with the control. Hydrogen peroxide disk assay was used to assay the survival rate of *E. coli* [46].

Cell survival rate after ionizing radiation was determined by the method described previously [47]. In short, after the cells were harvested, 200 µL of various MOxyR strains and wild type strains were irradiated at room temperature for 1h with ^60^Co γ-rays at various distances from the source, which correspond to doses from 0 to 24 kGy. After irradiation, the various MOxyR and wild type strains were plated on TGY plates and incubated at 30°C for 3 days prior to colony enumeration.

All the data provided here represent the mean and standard deviation of at least three independent experiments.

### Catalase activity assay

Whole-cell protein extracts were obtained from *D. radiodurans* cells in exponential phase growth according to the method of Tian *et al* [48]. Catalase activity was determined as in [49]: 2 µL of 0.2 mg/mL protein was diluted with 38 µL of PBS buffer, 10 µL of 250 mM H_2_O_2_ was then added followed by incubation at 25°C for 2 min. The reaction was quenched with 450 µL of 1% sodium azide. 10 µL of this mixture was diluted with 200 µL of an appropriate chromogenic reagent miture (Beyotime, CHN), in a 1-cm path-length polystyrene cuvette. The quantity of H_2_O_2_ remaining in the mixture can be determined by the oxidative coupling of 4-aminophenazone (4-aminoantipyrene, AAP) and 3,5-dichloro-2-hydroxybenzenesulfonic acid (DHBS) in the presence of H_2_O_2_ when catalyzed by horseradish peroxidase (HRP) contained in the chromogenic reagent mixture. After 15 min of incubation at 25°C, the resultant quinoneimine dye (N-(4-antipyrl)-3-chloro-5-sulfonate-p-benzoquinonemonoimine) was quantitated at 520 nm. H_2_O_2_ concentrations were determined by reference to a calibration curve generated from H_2_O_2_ solutions in the range 0–0.5 µM. The activity of catalase (expressed in micromoles of H_2_O_2_ decomposed per minute per milligram of total protein) was calculated as follows:
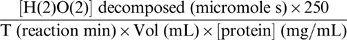



### Intracellular ROS accumulation assay

ROS generation in cells was assayed as reported [50]. In short, 10^7^ cells (100 µL, OD_600_ = 1.0, washed three times with PBS) were resuspended in 1 mL DCFH-DA (10 µM) and incubated at 37°C for 20 minutes. After incubation, the cells were washed twice with PBS and re-suspended in 1 mL PBS. Then, the sample was divided in two parts, half (0.5 mL) was exposed to 1 µL of 10M H_2_O_2_ for 20 minutes at room temperature, the other half served as the nonexposed control culture. The fluorescence intensities were measured using a fluorescence spectrophotometer with an excitation wavelength of 485 nm and an emission wavelength of 525 nm.

### Expression and purification of OxyR wild-type and mutant proteins

Wild-type *oxyR* was produced from pMD*oxyR*, which was digested with *Nde*I and *Bam*HI. Mutant *oxyR* was obtained from pMD18*oxyR*sd digested with *Nde*I and *Bam*HI. The products were ligated into pET28a (Novagen, San Diego, CA), and the resulting plasmids were transformed into *E. coli* BL21 for overexpression of His-tagged proteins, respectively. Protein expression and purification as previously described [22]. Briefly, an overnight culture was diluted 1: 50 in fresh media to an OD_600_ of 0.3 at 37°C and followed by a shift to 4°C. After 0.5 hours incubation at 4°C, cells were induced with 0.1 mM isopropyl-β-D-thiogalactoside (IPTG) for 15 hours at 20°C. The harvested cells were resuspended in lysis buffer (20 mM NaH_2_PO_4_; 20 mM Na_2_HPO_4_; 400 mM NaCl; 15 mM imidazole; 1 mM DTT) and disrupted at 4°C in a sonicator. After centrifugation at 12, 000 rpm for 20 min at 4°C, the supernatant was loaded onto Ni-NTA agarose columns (Qiagen, Valencia, CA). The purified OxyR protein was applied to a Superdex 300 HR 16/70. The purity of protein samples was determined using 12% SDS-PAGE, and only fractions with pure OxyR protein were used for further experiments.

### Reversible formation of cysteine-sulfenic acid trapping with NBD chloride

Modification of OxyR protein by NBD chloride (Sigma) at C210 was detected as previously described [8], [20], [39], [51]. Briefly, reduced or oxidized proteins were mixed with 1 mM NBD-Cl in dimethyl sulfoxide and incubated for 60 min at 25°C in the dark. Then the NBD-Cl was removed by ultrafiltration with YM-10 (Millipore, Bedford, MA) dialysed three times in 50 mM pH 7.0 potassium phosphate buffer containing 150 mM NaCl and 1 mM EDTA. The absorbance spectra (300 to 600 nm) of the modified proteins were measured on a ND-1000 spcetrophotometer (NanoDrop, Wilmingon, De. US).

### RNA isolation, quantitative real-time PCR (QRT-PCR) experiments

The wild-type strain and MOxyR strain were grown in TGY to mid-exponential phase. For H_2_O_2_ treatment, the cultures were divided in two; one half of the culture was treated with H_2_O_2 _at a final concentration of 20 mM, while the other half was used as non-treatment control. RNA isolation, and QRT-PCR were carried out as previously described [28]. Total RNA was extracted from cells using TRI Reagent (Invitrogen, Carlsbad, CA), after liquid nitrogen grinding. Then the RNA samples were treated with Rnase free Dnase I (Promega, Madison, WI) and purified using phenol chloroform extraction. RNA quality and quantity were evaluated by UV absorbance at 260 and 280 nm.

QRT-PCR assay utilized RNA samples obtained from different conditions and first-strand cDNA synthesis was carried out in 20 µL of reaction containing 1 µg of RNA sample combined with 3 µg of random hexamers using SuperScript III Reverse Transcriptase kit (Invitrogen). Then Quant SYBR Green PCR kit (TIANGEN, BJ) was used to amplification following the manufacturer's instructions. As an internal control, a house-keeping gene encoding glycosyl transferase, *dr0089*, was used as a house-keeping gene, encoding the glycosyl transferase [28]. In our hands, DR0089 was unaffected by any of our treatments. cDNA probes for microarray hybridization were prepared from four biological replicate total RNA samples each of wild type and MOxyR cultures. All primers used in QRT-PCR are shown in Table 2.

### Microarray hybridization and data analysis

Microarray design and constructions were carried out as our previous work [28]. Total RNA for microarray hybridization were obtained from four biological replicate samples of each of wild type and MOxyR under normal condition. First, RNA (4 µg) was annealed to 9 µg of random hexamer primers (Takara) in total volume of 20 µL at 70°C for 10 min and subsequent keep on ice for 2 min. cDNA was synthesized at 42°C overnight in total 31 µL using SuperScript III Reverse Transcriptase kit (Invitrogen) with 0.5 mM dNTP mix containing amino allyl-dUTP (GE, Piscataway, NJ). The reaction was terminated by adding 20 µL EDTA (0.5 M), and RNA was hydrolysed by adding 20 µL NaOH (1 M), then incubating at 65°C for 20 min. After neutralized with 50 µL Hepes (1M, pH 7.0), unincorporated free amino allyl-dUTPs were removed by ultrafiltration with YM 30 (Millipore), and resultant cDNA samples were coupled to 1 pmol Cy3 or Cy5 dyes (GE) in 0.1 M sodium carbonate buffer for 2 h at room temperature in the dark. Unincorporated free Cy3 or Cy5 were removed by ultrafiltration with YM 30. Two labeled cDNA pool (wild type and MOxyR) to be compared were mixed and hybridized simultaneously to the array in a solution containing 3×saline sodium citrate (SSC), 0.3 % SDS, and 24 µg of unlabeled herring sperm DNA (Gibco BRL, Gaithersburg, MD) [52]. Following hybridization, slides were washed as published paper [52].

Measurement of spot intensity and normalization were carried out as our paper [28]. In short, slides were scanned with a GenePix 4000B imager (Axon, Union City, CA), and spot intensities were obtained by software GenePix pro 5.1. Normalization and statistical analysis were carried out in the R computing environment (2.11, Raqua on the Windows) using the linear models for microarray data package (Limma) [53]. Within Limma, prior to channel normalization, microarray outputs were filtered to remove spots of poor signal quality by excluding those data points with mean intensity less than two standard deviations above background in both channels. Then, global LOESS normalization was used to normalize all data, and the 2-replicate spots per gene in each array were used to maximize the robustness of differential expression measurement of each gene via the “lmFit” function [54]. The microarray data have been deposited in the Gene Expression Omnibus database under accession no. GSE9636.

### Assay of intracellular Fe and Mn ion concentration

Total iron and Mn concentration were detected as described previously with some modification [55]. Bacteria were grown aerobically to OD600 0.8 in 600 ml of TGY broth. After centrifugation at 10,000 g for 10 min at 4°C, cells were washed twice in 200 ml of phosphate-buffered saline (PBS) with 1 mM EDTA (pH 7.4) and resuspended in 200 ml of PBS without EDTA. After centrifugation, the pellet was resuspended in 10 ml of PBS, 8 ml of which was used for iron and manganese analysis. Cell dry weight was estimated with the remaining 2ml suspension. For iron and manganese analysis, pelleted bacteria were resuspended in 1 ml of Ultrex II nitric acid (Fluka AG., Buchs, Switzerland) and incubated at 80°C for 1 hour. After centrifugation at 20,000 g for 20 minutes, the supernatant was filtered against 0.45 µM membrane. The concentration of samples was analyzed for iron and Mn content by inductively coupled plasma-optical emission spectroscopy (ICP-MS, Model Agilent 7500a, Hewlett-Packard, Yokogawa Analytical Systems, Tokyo, Japan). All buffers and nitric acid solutions were analyzed as described above to correct for background.

### Gel mobility shift assays

Gel mobility shift assays were performed with FITC-labeled DNA fragments (0.05 pmol) mixed with purified OxyR protein (oxidized protein or reduced protein, 200 nmol) in a total volume of 20 µL. The binding buffer contained 10 mM Tris-Cl (pH 8.0), 50 mM NaCl, 1 mM EDTA, 5% Glycerol, 50 µg/mL BSA and 5 µg/mL calf thymus DNA [22], [39]. The reaction mixture was incubated at room temperature for 30 min and loaded onto a 1.5% agarose gel in 0.5×TBE. Electrophoresis was performed at 80 V for 1 h at 4°C and followed by photographed [56], [57]. For generation of the labeled DNA, the appropriate operator fragment of target genes (*dr1709*, *dr1998*, *dr2263*, *drB0036*, and *drB0125*) was amplified by PCR with genomic DNA and cloned into pMD18, followed by FITC-labeled RV-M and reverse priming of target genes. In addition, *dr0207* was used as negative control. All primers are listed in Table 2.

## Supporting Information

Figure S1 H2O2 disk assay. Photos showing the zones of inhibition by H2O2 in E. coli K-12 (wild type) (A), GS09 (oxyR::kan mutant) (B), and E. coli K-12 strain GS09 complemented with the droxyR gene (C). (D) Histogram showing the results of H2O2 disk assay in E. coli. In the assay, E. coli cells were grown in Luria-Bertani (LB) broth at 37 °C with overnight shaking. 200 µl of the overnight cultures were added to LB top agar and spread onto LB agar. Then, 10 µl of 3% H2O2 was pipetted onto 3MM Whatman paper disks (0.7-cm diameter), and these disks were placed on top of the agar and incubated at 37 °C overnight. The zone of inhibition, in mm, was taken as a measure of H2O2 sensitivity. The zone of inhibition was measured in three dimensions, and the mean values and standard deviations were calculated.
(4.13 MB TIF)

Figure S2 Disruption of D. radiodurans droxyR gene. Verification of droxyR gene disruption by PCR analysis. Purified PCR fragments were amplified from the genomic DNA of strain R1 and MOxyR using primers (OxyR1 and OxyR5) that flank the coding sequences for droxyR. The PCR products of R1 revealed a band of ∼2550 bp length (band 1), whereas those of MOxyR resulted in a ∼3500 bp fragment (band 2). Bands 3 and 4 denote PCR products of R1 and MOxyR were digested with BamHI, respectively. Bands 5 and 6 denote PCR products of R1 and MOxyR were digested with HindIII, respectively. M denotes molecular weight standards.
(2.63 MB TIF)

Table S1 The repressed genes showed in MOxyR. All genes are sorted by fold induction or repression.
(0.08 MB DOC)

Table S2 The induced genes showed in MOxyR. All genes are sorted by fold induction or repression.
(0.09 MB DOC)

Table S3 Functional classification of genes with statistically significantly induction or repression in untreated wild-type strains compared to untreated oxyR mutant.
(0.03 MB DOC)

## References

[1] Imlay JA (2003). Pathways of oxidative damage.. Annu Rev Microbiol.

[2] Zheng M, Aslund F, Storz G (1998). Activation of the OxyR transcription factor by reversible disulfide bond formation.. Science.

[3] Christman MF, Morgan RW, Jacobson FS, Ames BN (1985). Positive control of a regulon for defenses against oxidative stress and some heat-shock proteins in Salmonella typhimurium.. Cell.

[4] Helmann JD (2002). OxyR: a molecular code for redox sensing?. Sci STKE.

[5] Tseng HJ, McEwan AG, Apicella MA, Jennings MP (2003). OxyR acts as a repressor of catalase expression in Neisseria gonorrhoeae.. Infect Immun.

[6] Lee C, Lee SM, Mukhopadhyay P, Kim SJ, Lee SC (2004). Redox regulation of OxyR requires specific disulfide bond formation involving a rapid kinetic reaction path.. Nat Struct Mol Biol.

[7] Toledano MB, Kullik I, Trinh F, Baird PT, Schneider TD (1994). Redox-dependent shift of OxyR-DNA contacts along an extended DNA-binding site: a mechanism for differential promoter selection.. Cell.

[8] Kim SO, Merchant K, Nudelman R, Beyer WF, Keng T (2002). OxyR: a molecular code for redox-related signaling.. Cell.

[9] Battista JR, Earl AM, Park MJ (1999). Why is Deinococcus radiodurans so resistant to ionizing radiation?. Trends Microbiol.

[10] Minton KW (1994). DNA repair in the extremely radioresistant bacterium Deinococcus radiodurans.. Mol Microbiol.

[11] Tanaka M, Narumi I, Funayama T, Kikuchi M, Watanabe H (2005). Characterization of pathways dependent on the uvsE, uvrA1, or uvrA2 gene product for UV resistance in Deinococcus radiodurans.. J Bacteriol.

[12] Earl AM, Rankin SK, Kim KP, Lamendola ON, Battista JR (2002). Genetic evidence that the uvsE gene product of Deinococcus radiodurans R1 is a UV damage endonuclease.. J Bacteriol.

[13] Markillie LM, Varnum SM, Hradecky P, Wong KK (1999). Targeted mutagenesis by duplication insertion in the radioresistant bacterium Deinococcus radiodurans: radiation sensitivities of catalase (katA) and superoxide dismutase (sodA) mutants.. J Bacteriol.

[14] Tanaka M, Earl AM, Howell HA, Park MJ, Eisen JA (2004). Analysis of Deinococcus radiodurans's transcriptional response to ionizing radiation and desiccation reveals novel proteins that contribute to extreme radioresistance.. Genetics.

[15] Ghosal D, Omelchenko MV, Gaidamakova EK, Matrosova VY, Vasilenko A (2005). How radiation kills cells: survival of Deinococcus radiodurans and Shewanella oneidensis under oxidative stress.. FEMS Microbiol Rev.

[16] Seib KL, Wu HJ, Srikhanta YN, Edwards JL, Falsetta ML (2007). Characterization of the OxyR regulon of Neisseria gonorrhoeae.. Mol Microbiol.

[17] Kullik I, Stevens J, Toledano MB, Storz G (1995). Mutational analysis of the redox-sensitive transcriptional regulator OxyR: regions important for DNA binding and multimerization.. J Bacteriol.

[18] Wang X, Mukhopadhyay P, Wood MJ, Outten FW, Opdyke JA (2006). Mutational analysis to define an activating region on the redox-sensitive transcriptional regulator OxyR.. J Bacteriol.

[19] Christman MF, Storz G, Ames BN (1989). OxyR, a positive regulator of hydrogen peroxide-inducible genes in Escherichia coli and Salmonella typhimurium, is homologous to a family of bacterial regulatory proteins.. Proc Natl Acad Sci U S A.

[20] Ellis HR, Poole LB (1997). Novel application of 7-chloro-4-nitrobenzo-2-oxa-1,3-diazole to identify cysteine sulfenic acid in the AhpC component of alkyl hydroperoxide reductase.. Biochemistry.

[21] Zheng M, Wang X, Templeton LJ, Smulski DR, LaRossa RA (2001). DNA microarray-mediated transcriptional profiling of the Escherichia coli response to hydrogen peroxide.. J Bacteriol.

[22] Zeller T, Mraheil MA, Moskvin OV, Li K, Gomelsky M (2007). Regulation of hydrogen peroxide-dependent gene expression in Rhodobacter sphaeroides: regulatory functions of OxyR.. J Bacteriol.

[23] Harrison A, Ray WC, Baker BD, Armbruster DW, Bakaletz LO (2007). The OxyR regulon in nontypeable Haemophilus influenzae.. J Bacteriol.

[24] Diaz PI, Slakeski N, Reynolds EC, Morona R, Rogers AH (2006). Role of oxyR in the oral anaerobe Porphyromonas gingivalis.. J Bacteriol.

[25] Kehres DG, Janakiraman A, Slauch JM, Maguire ME (2002). Regulation of Salmonella enterica serovar Typhimurium mntH transcription by H(2)O(2), Fe(2+), and Mn(2+).. J Bacteriol.

[26] Kim SG, Bhattacharyya G, Grove A, Lee YH (2006). Crystal structure of Dps-1, a functionally distinct Dps protein from Deinococcus radiodurans.. J Mol Biol.

[27] Cuypers MG, Mitchell EP, Romao CV, McSweeney SM (2007). The crystal structure of the Dps2 from Deinococcus radiodurans reveals an unusual pore profile with a non-specific metal binding site.. J Mol Biol.

[28] Chen H, Xu ZJ, Tian B, Chen WW, Hu SN (2007). Transcriptional profile in response to ionizing radiation at low dose in Deinococcus radiodurans.. Progress in Natural Science.

[29] Daly MJ, Gaidamakova EK, Matrosova VY, Vasilenko A, Zhai M (2004). Accumulation of Mn(II) in Deinococcus radiodurans facilitates gamma-radiation resistance.. Science.

[30] Bsat N, Herbig A, Casillas-Martinez L, Setlow P, Helmann JD (1998). Bacillus subtilis contains multiple Fur homologues: identification of the iron uptake (Fur) and peroxide regulon (PerR) repressors.. Mol Microbiol.

[31] Rocha ER, Herren CD, Smalley DJ, Smith CJ (2003). The complex oxidative stress response of Bacteroides fragilis: the role of OxyR in control of gene expression.. Anaerobe.

[32] Runyen-Janecky L, Dazenski E, Hawkins S, Warner L (2006). Role and regulation of the Shigella flexneri sit and MntH systems.. Infect Immun.

[33] Lander HM (1997). An essential role for free radicals and derived species in signal transduction.. Faseb J.

[34] Veal EA, Day AM, Morgan BA (2007). Hydrogen peroxide sensing and signaling.. Mol Cell.

[35] Liu Y, Zhou J, Omelchenko MV, Beliaev AS, Venkateswaran A (2003). Transcriptome dynamics of Deinococcus radiodurans recovering from ionizing radiation.. Proc Natl Acad Sci U S A.

[36] Harris DR, Tanaka M, Saveliev SV, Jolivet E, Earl AM (2004). Preserving genome integrity: the DdrA protein of Deinococcus radiodurans R1.. PLoS Biol.

[37] Xu Z, Tian B, Sun Z, Lin J, Hua Y (2007). Identification and functional analysis of a phytoene desaturase gene from the extremely radioresistant bacterium Deinococcus radiodurans.. Microbiology.

[38] Daly MJ, Gaidamakova EK, Matrosova VY, Vasilenko A, Zhai M (2007). Protein oxidation implicated as the primary determinant of bacterial radioresistance.. PLoS Biol.

[39] Fuangthong M, Helmann JD (2002). The OhrR repressor senses organic hydroperoxides by reversible formation of a cysteine-sulfenic acid derivative.. Proc Natl Acad Sci U S A.

[40] Lee JW, Soonsanga S, Helmann JD (2007). A complex thiolate switch regulates the Bacillus subtilis organic peroxide sensor OhrR.. Proc Natl Acad Sci U S A.

[41] Du X, Takagi H (2007). N-Acetyltransferase Mpr1 confers ethanol tolerance on Saccharomyces cerevisiae by reducing reactive oxygen species.. Appl Microbiol Biotechnol.

[42] Wallecha A, Correnti J, Munster V, van der Woude M (2003). Phase variation of Ag43 is independent of the oxidation state of OxyR.. J Bacteriol.

[43] Gao GJ, Lu HM, Huang LF, Hua YJ (2005). Construction of DNA damage response gene pprI function deficient and function complementary mutants in Deinococcus radiodurans.. Chinese Science Bulletin.

[44] Hua Y, Narumi I, Gao G, Tian B, Satoh K (2003). PprI: a general switch responsible for extreme radioresistance of Deinococcus radiodurans.. Biochem Biophys Res Commun.

[45] Brenot A, King KY, Caparon MG (2005). The PerR regulon in peroxide resistance and virulence of Streptococcus pyogenes.. Mol Microbiol.

[46] Raahave D (1974). Paper disk-agar diffusion assay of penicillin in the presence of streptomycin.. Antimicrob Agents Chemother.

[47] Huang L, Hua X, Lu H, Gao G, Tian B (2007). Three tandem HRDC domains have synergistic effect on the RecQ functions in Deinococcus radiodurans.. DNA Repair (Amst).

[48] Tian B, Wu Y, Sheng D, Zheng Z, Gao G (2004). Chemiluminescence assay for reactive oxygen species scavenging activities and inhibition on oxidative damage of DNA in Deinococcus radiodurans.. Luminescence.

[49] Esposito P, Varvara G, Caputi S, Perinetti G (2003). Catalase activity in human healthy and inflamed dental pulps.. Int Endod J.

[50] Kang SW, Baines IC, Rhee SG (1998). Characterization of a mammalian peroxiredoxin that contains one conserved cysteine.. J Biol Chem.

[51] Panmanee W, Vattanaviboon P, Poole LB, Mongkolsuk S (2006). Novel organic hydroperoxide-sensing and responding mechanisms for OhrR, a major bacterial sensor and regulator of organic hydroperoxide stress.. J Bacteriol.

[52] Thompson DK, Beliaev AS, Giometti CS, Tollaksen SL, Khare T (2002). Transcriptional and proteomic analysis of a ferric uptake regulator (fur) mutant of Shewanella oneidensis: possible involvement of fur in energy metabolism, transcriptional regulation, and oxidative stress.. Appl Environ Microbiol.

[53] Wettenhall JM, Smyth GK (2004). limmaGUI: a graphical user interface for linear modeling of microarray data.. Bioinformatics.

[54] Smyth GK, Michaud J, Scott HS (2005). Use of within-array replicate spots for assessing differential expression in microarray experiments.. Bioinformatics.

[55] Ma JF, Ochsner UA, Klotz MG, Nanayakkara VK, Howell ML (1999). Bacterioferritin A modulates catalase A (KatA) activity and resistance to hydrogen peroxide in Pseudomonas aeruginosa.. J Bacteriol.

[56] Brune I, Werner H, Huser AT, Kalinowski J, Puhler A (2006). The DtxR protein acting as dual transcriptional regulator directs a global regulatory network involved in iron metabolism of Corynebacterium glutamicum.. BMC Genomics.

[57] Timmins J, Leiros I, McSweeney S (2007). Crystal structure and mutational study of RecOR provide insight into its mode of DNA binding.. Embo J.

[58] Choi H, Kim S, Mukhopadhyay P, Cho S, Woo J (2001). Structural basis of the redox switch in the OxyR transcription factor.. Cell.

[59] Anderson AW, Nordon HC, Cain RF, Parrish G, Duggan G (1956). Studies on a radio-resistant micrococcus. I. Isolation, morphology, cultural characteristics, and resistance to gamma radiation.. Food Technol.

