# Supplementary material for: A Novel OxyR Sensor and Regulator of Hydrogen Peroxide Stress with One Cysteine Residue in Deinococcus radiodurans
Source: PLoS One. 2008 Feb 13;3(2):e1602. doi: 10.1371/journal.pone.0001602 (PMC2225504; doi:10.1371/journal.pone.0001602)
Supplement: Supplementary file 1 [file pone.0001602.s003.doc]

Table S1. The repressed genes showed in MOxyR. All genes are sorted by fold induction or repression.

Locus	Annotation	M	P
DR0096	ABC Transporter with ATPase domain	-5.12704	2.68E-05
DR1440	Cation-transporting ATPase, authentic frameshift	-4.74244	0.000119
DRA0157	Periplasmic phosphate-binding protein PSTS	-4.47856	0.000648
DR2209	Rio1 family Protein kinase	-4.42562	5.80E-05
DR1263	Protein of uncharacterized conserved family predominantly in C elegansi	-4.38007	0.001641
DRB0067	Extracellular nuclease with Fibronectin III domains	-3.63096	4.75E-06
DR0095	ABC Transporter with ATPase domain	-3.53127	7.25E-05
DRA0019	NH2-acetyltransferase	-3.19338	0.002963
DRA0159	Phosphate transport permease PSTA	-3.06378	2.35E-05
DR1624	RNA helicase, authentic frameshift	-3.00083	2.01E-05
DR2409	McrA family nuclease	-2.65937	0.00069
DR0548	Pilin IV like secreted protein, most similar to leader peptidase IV, TAPA	-2.63356	1.20E-06
DR0353	VacB, S1 domain nuclease	-2.62564	5.28E-05
DR1978	NH2-acetyltransferase family	-2.59336	0.003969
DR1998	Catalase, CATX	-2.59086	0.000272
DRA0020	COBU cobinamide kinase/cobinamide phosphate guanylyltransferase	-2.47193	0.00179
DRA0017	Thioesterase	-2.4306	0.00152
DR2449	Small conserved bacterial proteins, YOHL_ECOLI	-2.37426	0.00625
DR2339	LigT, 2'-5' RNA LIGASE	-2.37082	0.001245
DR0352	Predicted protein	-2.30813	0.000498
DR2441	NH2-acetyltransferase	-2.274	0.000183
DR0396	Predicted protein	-2.21505	0.000548
DR2453	P-type ATPase metal efflux	-2.18394	0.00816
DR0533	Predicted protein	-2.13101	0.001366
DR1630	Conserved transport protein	-2.10621	0.012968
DR1583	Conserved membrane protein	-2.08896	0.000343
DR1200	Predicted protein	-2.02212	0.003261
DR2385	Phenylacetic acid degradation protein PaaB	-2.01609	0.000759
DR0746	Predicted protein	-2.0116	0.00016
DR2208	Glyoxalase/Dioxygenase family protein	-1.98719	0.01098
DR1976	MutS- Mismatch repair, ABC superfamily ATPase	-1.97696	0.00046
DR1419	P-loop containing protein, tunicamycin resistance protein ortholog	-1.9646	0.011378
DR2384	Phenylacetic acid degradation protein PaaC	-1.95686	3.92E-05
DR1582	Conserved membrane protein	-1.9147	0.000821
DR0203	Membrane protein, similar to gi|1653436 of Synechocystis	-1.89917	0.022306
DR1907	Fe-S subunit of glycolate oxidase, YKGE	-1.89843	0.007343

DR2424	Transposase, putative, authentic frameshift	-1.8642	0.014134
DR1057	NH2- Acetyltransferase	-1.85872	0.002539
DR2617	Cytochrome AA3 controlling protein, ctaA	-1.83778	0.013982
DRB0068	Secreted protein with Fibronectin III domains	-1.83031	0.001742
DRA0158	Phosphate transport system permease PSTA	-1.82102	0.006286
DR2383	MinD N- terminal domain, PaaD ortholog	-1.81413	0.002136
DR0070	Predicted protein	-1.75259	0.00157
DR0265	HTH transcription factor, GntR family	-1.73751	0.001003
DR0763	Probable N-acetyltransferase	-1.72881	0.015328
DR0234	Predicted protein	-1.72577	0.012119
DR2338	CinA ortholog, CinA MoeB family	-1.7181	0.000185
DR0139	OBG family GTPase	-1.71521	0.001565
DRA0257	Ankyrin repeat protein	-1.71139	0.00134
DRB0106	Acyl-CoA Thioesterase superfamily protein	-1.67655	0.000486
DR2452	Metal-binding subunit of ATPase metal efflux	-1.674	0.003755
DR2574	HTH transcription factor, phage type	-1.66145	0.000806
DR0019	FTSZ fragment	-1.6547	0.004408
DR0821	Acetylcholinesterase family protein	-1.64704	0.000738
DRC0019	Transposase	-1.63941	0.007761
DR1927	Transposase	-1.62917	0.02012
DR2451	Uncharacterized conserved protein containing duplicated domain with conserved HH motif	-1.6253	0.000907
DR0905	Uncharacterized protein similar to YGY4_HALSQ	-1.59605	0.006664
DRB0116	Predicted protein	-1.59156	0.021562
DR1710	Predicted protein	-1.58968	0.011425
DR0346	Cytochrome C-Type biogenesis protein CYCK	-1.54709	0.002106
DR0204	Membrane protein, similar to gi|1653967 of Synechocystis	-1.51905	0.002845
DR0133	Lipase, a/b superfamily hydrolase	-1.51018	0.003864
DR2450	Predicted protein	-1.50172	0.001483
DR1581	ABC ATPase, MDR type	-1.49726	0.000463
DR1906	L-lactate permease	-1.46065	4.23E-05
DR1772	Predicted protein	-1.45875	0.027799
DR2308	OBG family GTPase	-1.44114	0.002517
DR1578	Uncharacterized enzyme	-1.43832	0.002176
DR0985	Uncharacterized protein, bacillus YACC ortholog	-1.42953	0.040915
DR1493	NADH DEHYDROGENASE subunit M (NQO13)	-1.42658	0.005112
DR1292	Molybdenum cofactor biosynthesis protein B, MoaB protein	-1.41319	0.03044
DR0663	Predicted protein	-1.40573	0.003272
DR2391	Von-willebrand-factor A domain containing protein	-1.39864	0.010663
DRB0104	XerC-like recombinase	-1.39762	0.007296
DRA0258	Predicted protein	-1.38724	0.006715
DR1161	Transthyretin	-1.36976	0.001621

DR2273	Probable P-loop containing kinase, FlaR-like protein	-1.35361	0.004584
DR1160	Uricase	-1.34384	0.000961
DR1799	Initiation factor IF-2	-1.34066	0.002127
DR0342	Iron sulfur protein, Rieske type	-1.3297	0.020873
DR2470	Conserved membrane protein, related to biothin biosysnthesis protein	-1.32586	0.027048
DR1264	Predicted protein	-1.31832	0.037004
DR1505	NADH DEHYDROGENASE subunit B (NQO6)	-1.31695	0.037111
DRB0051	Predicted protein	-1.3069	0.037905
DR2524	Ribosomal protein L28	-1.30687	0.005166
DR0003	Predicted protein	-1.29379	0.012489
DR1996	Fatty acid/phospholipid synthesis protein PlsX, authentic frameshift	-1.27542	0.005167
DR2051	ABC Transporter ATPase, MDR-type	-1.27332	0.003207
DR2618	Cytochrome c oxidase assembly factor, ctaB	-1.27331	0.000896
DR0046	L-rich repeats containing protein	-1.2663	0.041338
DR1558	Receiver domain (flavodoxin, CHEY family) of 2 component system + C-terminal HTH	-1.24785	0.043023
DR0906	DNA gyrase B subunit, GYRB	-1.24773	0.005522
DR1190	Conserved membrane protein	-1.24692	0.023465
DRB0069	Subtilisin serine protease	-1.24029	0.000565
DR1359	NlpA like Lipoprotein	-1.2352	0.002205
DR2374	Ribonucleotide reductase, archeal type	-1.2309	0.013401
DR0126	in, authentic frameshift	-1.2276	0.002258
DRB0107	NRDI ribonucleoprotein	-1.22526	0.002171
DRB0111	Glycerophosphodiester phosphodiesterase	-1.20099	0.009128
DR2340	RecA	-1.19142	0.007686
DR0205	ABC transporter ATPase	-1.1875	0.019387
DR0438	Predicted protein	-1.18712	0.004462
DR1575	Predicted protein	-1.18464	0.049463
DR1482	2-isopropylmalate synthase, LEU1	-1.16947	0.000901
DR2439	Yfit/DinB family protein	-1.16782	0.001723
DR0343	Cytochrome oxidase family protein	-1.15833	0.007039
DR1796	Small highly conserved protein always associated with the NusA gene	-1.1567	0.002023
DR2427	Shares a domain with YjiI	-1.1556	0.004486
DR0292	Conserved secreted small protein related to TatA/TatB of e.coli.	-1.15033	0.017964
DR2381	MaoC like aldehyde dehydrogenase, PaaZ	-1.11905	0.007563
DR1995	Conserved membrane protein	-1.1132	0.003712
DR1904	Predicted protein	-1.10424	0.004347
DR1356	ABC transporter, ATP-binding protein	-1.09887	0.013557
DRA0248	Transcriptional regulator, MarR/SlyA family	-1.09256	0.011031
DR1577	QueA ortholog, S-adenosylmethionine:trna ribosyltransferase-isomerase	-1.08947	0.000535
DR0460	Acetyl-CoA synthetase	-1.07283	0.011562
DR1189	Methylmalonyl-CoA mutase	-1.06786	0.001949

DR0127	Ankyrin repeats related protein	-1.04611	0.023765
DR0156	Riboflavin synthase beta subunit, RisB	-1.04114	0.034828
DR1797	NusA	-1.03718	0.005014
DR1118	PilT N-terminal domain like protein, merged with transmembrane segments	-1.03345	0.016045
DR2115	Ribosomal protein L15	-1.02713	0.010582
DR2440	YohI/YHDG family of TIM barrel dinucleotide binding enzymes	-1.0183	0.013943
DR2386	MaoC like aldehyde dehydrogenase PaaA	-1.01801	0.004797
DR1198	TYPA like GTPase	-1.01161	0.022671
DR2225	Tellurium resistance/ dictyostelium cAMP binding family of proteins	-1.00289	0.001629
DR2243	PhoU ortholog	-1.0013	0.019722
DR2078	Extracellular lipase, a/b fold hydrolase	-1.00129	0.010627
DR0053	DinB/YfiT family of proteins	-1.00048	0.033451
