# Supplementary material for: A Novel OxyR Sensor and Regulator of Hydrogen Peroxide Stress with One Cysteine Residue in Deinococcus radiodurans
Source: PLoS One. 2008 Feb 13;3(2):e1602. doi: 10.1371/journal.pone.0001602 (PMC2225504; doi:10.1371/journal.pone.0001602)
Supplement: Supplementary file 2 [file pone.0001602.s004.doc]

Table S2. The induced genes showed in MOxyR. All genes are sorted by fold induction or repression.

Locus	Annotation	M	P
DRA0211	HTH transcriptional regulator, GntR family	3.675916	0.001481
DR0959	ABC transporter permease dipeptide transporter?	3.615868	6.45E-05
DRA0154	Glutamine-fructose-6-phosphate transaminase, GLMS	3.518807	0.001715
DR1930	Membrane protein	3.14466	0.002494
DR1317	Predicted protein	2.975583	0.000207
DR1312	Nuclease McrA superfamily	2.847438	0.003462
DRA0343	Succinic semialdehyde dehydrogenase	2.807892	0.001363
DR2576	DHH family phosphohydrolase	2.662988	0.00431
DR1790	Yellow/royal jelly protein of insects	2.621214	0.000236
DR1901	Predicted protein	2.61182	0.004591
DR1225	Glycosyltransferase	2.530316	0.005089
DR2006	Predicted protein	2.45666	0.005599
DRB0017	Vibriobactin utilization protein viub	2.45529	0.005609
DR1385	Predicted protein	2.413336	0.00164
DR1809	Glycine dehydrogenase	2.408102	6.59E-05
DR2181	RAB/RAS like small bacterial GTPase	2.407659	0.00012
DR1231	Predicted protein	2.402131	0.001626
DR1811	Glycine cleavage system H protein	2.335541	0.001147
DR2179	Predicted protein	2.306999	3.11E-05
DRA0064	Thermostable? alkaline proteinase	2.294918	0.006969
DRA0245	Predicted protein	2.202938	0.007939
DR0645	MobA	2.179391	0.008214
DRA0192	Predicted protein	2.142951	0.008664
DRA0333	Zn-finger + FHA domain containing protein	2.099047	0.000166
DRB0036	Oxidoreductase, authentic point mutation	2.057709	0.015962
DRA0230	Predicted protein	2.017625	0.000262
DRA0331	VWFA Mg-binding domain protein	1.990624	0.000166
DR0995	Protein of uncharacterized conserved family	1.949141	0.008895
DR2182	Predicted protein	1.945342	0.003993
DR2180	Uncharacterized small family of predominantly archaeal proteins	1.921042	0.00011
DR2249	Protein phosphatase, calcineurin like phosphoesterase	1.909353	0.01243
DRA0334	Serine/Threonine kinase	1.876213	0.000141
DR1623	Predicted protein	1.869936	0.016431
DR1620	3-ketoacyl-acyl carrier protein reductase	1.867602	0.000962
DR0751	Cell division topological specificity factor, MinE	1.836379	0.000263
DRA0335	Serine/threonine protein kinase	1.835669	0.000293
DR1202	Predicted protein	1.805076	0.014771
DR1222	Acetyl-xylan esterase, NODB-like deacetylase	1.798468	0.014937

DR0251	Probable ribose/galactose ABC transporter	1.789405	0.015169
DR1844	NH2-acetyltransferase	1.785362	0.001144
DR1185	Surface protein	1.777477	0.000718
DR0894	Predicted protein	1.769102	0.002955
DR1944	Predicted protein	1.73132	0.000206
DR0105	LEA76 family desiccation resistance protein	1.713718	0.000177
DR1323	TRNA pseudouridine synthase TruB	1.712974	0.017314
DR1717	Racemase, L-carnitine Dehydratase family enzyme	1.709796	0.037424
DR1348	Predicted protein	1.68558	0.000642
DR1179	HKD superfamily hydrolase	1.684324	0.000231
DR1943	3-oxoacyl-acyl-carrier-protein reductase, FabG	1.657072	0.00019
DR1340	Uncharacterized protein	1.656865	0.003103
DR1596	Glucose-6-phosphate 1-dehydrogenase	1.652452	0.000527
DR2407	Predicted membrane protein, uncharacterized	1.647071	0.000399
DR1755	Predicted protein	1.627818	0.002376
DR2553	Sugar kinase (Ribokinase RbsK superfamily), fructokinase	1.621909	0.020389
DRA0007	NODI ABC ATPase	1.610955	0.020803
DRB0014	Periplasmic binding protein HutB	1.606685	0.005902
DR0274	MutT family hydrolase	1.604824	0.021039
DR1232	Pilin IV like secreted protein	1.594457	0.010676
DR0709	UDP-glucose 4-epimerase	1.586616	0.021761
DR0998	MinD family ATpase, MRP	1.573951	0.022282
DR1162	Transcription elongation factor GreA	1.563238	0.002249
DR0854	Small family of predominantly archaeal proteins	1.560039	0.001899
DR0442	PilT protein, ATPase	1.556802	0.000983
DR1203	N-term. domain of lmbE family protein, chracteristic HPDD motif	1.551107	0.02326
DR0201	Predicted protein	1.548435	0.00141
DR1812	Aminomethyltransferase, glycine cleavage system T protein	1.538327	0.015886
DR2559	Predicted protein	1.521312	0.001072
DR1708	Predicted protein	1.51489	3.49E-05
DR1067	Predicted protein	1.506497	0.000906
DR2240	Predicted protein	1.494368	0.001259
DR1697	Predicted protein	1.492888	0.006795
DR1595	6-phosphogluconate dehydrogenase	1.462784	0.000652
DR1897	Predicted protein	1.455628	0.000552
DR1616	Uncharacterized conserved membrane	1.453089	0.00048
DR1314	Uncharacterized proteins, ysnf-like repeats	1.424399	0.000105
DRB0032	Predicted protein	1.423798	0.029799
DRB0027	Fusion anti-sigma B factor RSBT/TORS + RSBX regulator	1.413582	9.66E-05
DR0407	Membrane protein related to cytochrome c maturation protein B	1.405868	0.030893
DRA0065	Chromosomal protein HU HupA	1.399851	0.01102
DR1804	Solo Double stranded beta helix protein, curC	1.397082	0.000896

DR0413	Predicted protein	1.395186	0.024074
DR1963	PilT-like ATPase	1.394994	0.010459
DR1987	Predicted protein	1.390382	0.000202
DR0399	Triple fascilin-like repeat, possible cell surface protein	1.382084	0.00126
DRA0164	Thioredoxin	1.370889	0.033174
DR0644	Predicted protein	1.370862	0.001019
DR0422	SAM dependent methyltransferase	1.370377	0.033209
DR1896	Predicted protein	1.368249	0.002502
DR0371	Predicted protein	1.363283	0.007263
DR0923	Uncharacterized ortholog of Mycobacterial and Cyanobacterial proteins	1.358518	0.03403
DR2433	Protein of nitrilase/amidohydrolase family	1.356035	0.034205
DRA0354	Similar to methyl-accepting chemotaxis protein PIL operon?	1.321465	0.017302
DR1803	Predicted protein	1.319007	0.048071
DRA0194	a/b hydrolase	1.311463	0.008746
DR0891	Receiver domain, CHEY family + HTH domain	1.311026	0.001034
DRA0364	ADG Oxidoreductase	1.310185	0.001306
DRA0352	PILJ ortholog, similar to methyl-accepting chemotaxis protein	1.310137	0.005147
DR1214	Acetyl-CoA carboxylase alpha subunit, ACCA	1.309377	0.00701
DR0972	Uncharacterized secreted protein	1.303353	0.001052
DR1945	Malonyl Acyl Carrier Transcyclase, FabD	1.298238	0.003143
DRA0005	NAD alcohol dehydrogenase	1.291897	0.039132
DR1341	Diverged aldoketomutase of glyoxalase I family	1.275152	0.00118
DR1571	Oligopeptide binding protein; oligopeptide transporter OppA	1.271434	0.008638
DR1893	Cyclophilin, peptidyl-prolyl isomerase	1.270096	0.000116
DR1146	GSP26 general stress like protein	1.266566	0.001413
DRC0007	Diverged PP-loop ATPase related to ExsB, 4 conserved C	1.264754	0.041477
DR1066	Predicted protein	1.262731	0.026503
DR2344	Predicted protein	1.260203	2.42E-06
DR2097	Predicted protein	1.259693	0.041932
DR1890	Rossman fold oxidoreductase	1.249228	0.001197
DR1768	Predicted protein	1.249179	0.000375
DR1837	Predicted protein	1.234899	0.044256
DR1879	Conserved membrane protein	1.231965	0.005824
DR0593	Alpha/beta hydrolase superfamily protein	1.228318	0.020911
DR1383	Predicted protein	1.22819	0.001289
DR1199	Protease I, related to general stress protein 18, ThiJ superfamily protein	1.222644	0.008486
DR1172	Lea76/LEa29-like desiccation resistance protein	1.219626	7.70E-05
DR0632	Related to YAFD_ECOLI	1.210994	0.046645
DR1331	Predicted protein	1.207284	0.002401
DRA0036	Possible membrane protein	1.200665	0.000199
DR2406	HGG containing thioesterase superfamily	1.190824	0.004703
DR1306	Predicted protein	1.182983	0.007313

DR2070	Conserved lipoprotein	1.180532	0.04992
DR0716	Nitrate reductase	1.170034	0.004083
DR2234	Diacylglycerol Kinase family protein	1.165569	0.044406
DR0615	HTH transcriptional regulator, LysR family	1.158116	0.005139
DR2596	Possible metalloprotease	1.136731	0.00928
DR2527	Predicted protein	1.129644	0.002913
DR1597	OPCA, OxPPCycle gene, involved in the assembly of above protein	1.125841	0.002286
DR1290	Periplasmic peptide binding protein	1.125266	0.004846
DR0781	Receiver domain, CHEY family + HTH domain	1.121185	0.000696
DR0711	UDP-glucose-4-epimerase	1.118734	0.042181
DR2188	Aminopeptidase T, AMP2	1.115389	0.006424
DR0685	Uncharacterized secreted protein	1.101534	0.000489
DR1980	NADH dehydrogenase, Rossman fold oxidoreductase	1.100141	0.00147
DR0845	Ribose 5-phosphate isomerase, RPIA	1.097495	0.001756
DR1874	GMP synthase, PP-loop ATPase	1.086703	0.013496
DR2310	Predicted protein	1.084224	0.005503
DR1361	Bacterial DCSG sequence containing hydrolase	1.079959	0.000303
DR2438	Endonuclease III, alpha helical glycosidase superfamily	1.071743	0.002349
DR0106	Predicted protein	1.070854	0.004271
DRB0054	Predicted protein	1.061976	0.005449
DR1840	Predicted protein	1.056885	0.005359
DR1253	Predicted protein	1.035494	0.011229
DR1220	HTH repressor FeoA	1.035212	0.015288
DR1662	Threonine 3-dehydrogenase	1.03423	0.002452
DR1215	Acetyl-CoA carboxylase beta subunit, ACCD	1.028654	0.008965
DRA0037	Glycosyltransferase	1.014303	0.001305
DR0888	Some domain + invasin domain protein	1.010724	0.003399
DR2235	PHP family phosphoesterase	1.006371	0.033357
