# Supplementary material for: A Novel OxyR Sensor and Regulator of Hydrogen Peroxide Stress with One Cysteine Residue in Deinococcus radiodurans
Source: PLoS One. 2008 Feb 13;3(2):e1602. doi: 10.1371/journal.pone.0001602 (PMC2225504; doi:10.1371/journal.pone.0001602)
Supplement: Supplementary file 3 [file pone.0001602.s005.doc]

Table S3. Functional classification of genes with statistically significantly induction or repression in untreated wild-type strains compared to untreated *oxyR* mutant.

Functional categories[1]	Induced number [2]	Repressed number [2]	Total
Energy production and conversion	5	8	13
Cell division and chromosome partitioning	2	1	3
Amino acid transport and metabolism	10	2	12
Nucleotide transport and metabolism	1	3	4
Carbohydrate transport and metabolism	8	0	8
Coenzyme metabolism	1	1	2
Lipid metabolism	9	7	16
Translation, ribosomal structure and biogenesis	1	7	8
Transcription	6	7	13
DNA replication, recombination and repair	2	8	10
Cell envelope biogenesis, outer membrane	7	1	8
Cell motility and secretion	5	0	5
Posttranslational modification, protein turnover, chaperones	4	5	9
Inorganic ion transport and metabolism	3	10	13
Secondary metabolites biosynthesis, transport and	5	2	7
General function prediction only	26	18	44
Function unknown	12	12	24
Signal transduction mechanisms	8	4	12
Hypothetical proteins	57	36	93

[1]Functional categories are based on the annotation of NCBI. [2]Expression changes are regarded as significant (fold change > 2 and $p < 0.05$). [3]The number of genes are involved in every COG category.
